# Mechanistic basis of the increased methylation activity of the SETD2 protein lysine methyltransferase towards a designed super-substrate peptide

**DOI:** 10.1038/s42004-022-00753-w

**Published:** 2022-10-28

**Authors:** Philipp Schnee, Michel Choudalakis, Sara Weirich, Mina S. Khella, Henrique Carvalho, Jürgen Pleiss, Albert Jeltsch

**Affiliations:** 1 Institute of Biochemistry and Technical Biochemistry, University of Stuttgart, Allmandring 31, 70569 Stuttgart, Germany; 2 Biochemistry Department, Faculty of Pharmacy, Ain Shams University, African Union Organization Street, Abbassia, Cairo, 11566 Egypt

**Keywords:** Transferases, Peptides, Chemical modification, Enzyme mechanisms, Computational chemistry

## Abstract

Protein lysine methyltransferases have important regulatory functions in cells, but mechanisms determining their activity and specificity are incompletely understood. Naturally, SETD2 introduces H3K36me3, but previously an artificial super-substrate (ssK36) was identified, which is methylated >100-fold faster. The ssK36-SETD2 complex structure cannot fully explain this effect. We applied molecular dynamics (MD) simulations and biochemical experiments to unravel the mechanistic basis of the increased methylation of ssK36, considering peptide conformations in solution, association of peptide and enzyme, and formation of transition-state (TS) like conformations of the enzyme-peptide complex. We observed in MD and FRET experiments that ssK36 adopts a hairpin conformation in solution with V35 and K36 placed in the loop. The hairpin conformation has easier access into the active site of SETD2 and it unfolds during the association process. Peptide methylation experiments revealed that introducing a stable hairpin conformation in the H3K36 peptide increased its methylation by SETD2. In MD simulations of enzyme-peptide complexes, the ssK36 peptide approached TS-like structures more frequently than H3K36 and distinct, substrate-specific TS-like structures were observed. Hairpin association, hairpin unfolding during association, and substrate-specific catalytically competent conformations may also be relevant for other PKMTs and hairpins could represent a promising starting point for SETD2 inhibitor development.

## Abstract (web summary)

The SET-domain containing protein 2 (SETD2) is a protein lysine methyltransferase that has been shown to methylate the super-substrate peptide ssK36 100 times faster than the corresponding natural H3K36 peptide. Here, a combination of molecular dynamics simulations and biochemical experiments are applied to identify the mechanisms responsible for the increased activity of SETD2 towards ssK36.

## Introduction

The methylation of histone and non-histone proteins, mainly at lysine and arginine residues, plays a key role in epigenetic regulation and cell signalling^1–3^. Histone lysine methylation has activating or repressive functions in the chromatin, depending on the position of the modified residue, the methylation state and the combination with other modifications^4^. Enzymes setting this modification are called protein lysine methyltransferases (PKMTs). One large family of PKMTs contains a conserved ~130 amino-acid SET (Su(var) 3-9, Enhancer of Zeste (E(z)) and Trithorax (trx)) domain, which forms the active site of the protein^5^. In the SET domain, the coenzyme S-adenosyl-L-methionine (SAM), which provides the activated methyl group, and the peptide containing the substrate lysine residue are located at opposing faces^6^. The lysine sidechain is bound into a hydrophobic tunnel allowing the ε-amino group of the lysine residue to approach the SAM methyl group in a deprotonated state^7^, eventually leading to a transfer of the methyl group in a bimolecular nucleophilic substitution (S_N_2) mechanism^5,8^.

The SET-domain containing protein 2 (SETD2, also called KMT3A) was first identified as huntingtin interacting protein 1 (HYPB, HIP-1)^9^. It has a size of 230 kDa corresponding to 2564 amino acids. SETD2 is the only PKMT known to introduce up to three methyl groups to lysine 36 of histone H3 (H3K36) in human cells^10,11^. H3K36me3 is enriched in the gene bodies of expressed genes since SETD2 is recruited by the elongating RNA Polymerase II^9^. Previous structural and kinetic studies have shown that an altered interaction of SETD2 with its peptide substrate can lead to strong biological effects. The K36M and K36I oncohistone mutants in histone variant H3.3 inhibit the methyltransferase activity of SETD2 because the methionine or isoleucine residue binds into the lysine binding tunnel of the active centre and thereby functions as a competitive enzyme inhibitor^12,13^. The inhibition of H3K36 PKMTs by the K36M/I oncohistones leads to massive perturbations of genome-wide H3K36me3 and H3K27me3 patterns and is frequently found in chondroblastomas^14^. Moreover, SETD2 frequently contains inactivating mutations in cancers^15^ and loss of SETD2 leads to global changes in DNA methylation patterns^16^.

Different structures of SETD2 in complex with H3K36 histone tail peptides and S-adenosyl-L-homocysteine (SAH) revealed that the peptide substrate is positioned in an extended conformation in a deep binding cleft in the catalytic SET domain of SETD2 where several contacts between the enzyme and the substrate peptide are formed^17–19^. Structure comparisons showed that the post-SET loop of SETD2 (amino acids 1692–1703) displays a dynamic behaviour that regulates autoinhibition^17^. In the state with no peptide bound in the SETD2 binding cleft, the post-SET loop is not resolved suggesting a flexibel behavior. Additionally, the autoinhibitory loop adopts a closed conformation and inserts R1670 into the active centre. This residue apparently functions as a placeholder and overlaps with H3K36M peptide in the SETD2-peptide complex, occupying the K36 access tunnel. An autoinhibitory function of equivalent loops was also observed for other PKMTs with different placeholder residues, such as serine in ASH1L or cysteine in NSD1^20^. In crystal structures of PKMTs with bound peptide, the post-SET loop was observed in  half opened and closed conformations covering the peptide in the active centre in a lid-like manner with the placeholder residues turned outwards. This indicates a dynamic opening and closing of this loop upon peptide binding and release^17^.

**Fig. 1:**
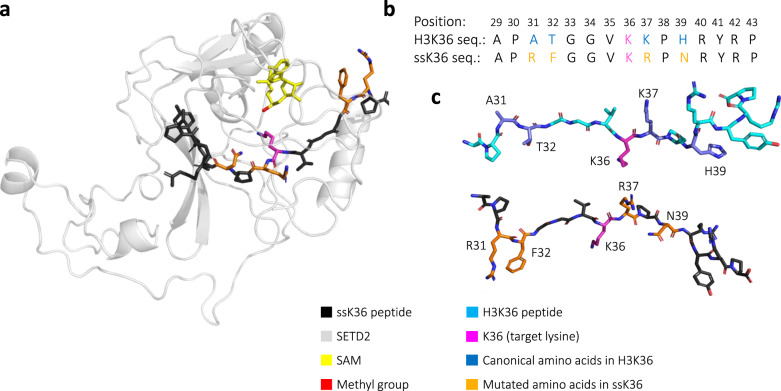
Model of the designed super-substrate peptide (ssK36). **a** Structure of ssK36 bound to SETD2 and SAM derived from PDB: 6VDB^19^. The ssK36 peptide was positioned in the active site of SETD2 with the target lysine reaching towards the SAM methyl group C-atom. A corresponding model was developed for H3K36 bound to SETD2 based on PDB 5V21^17^. **b** ssK36 differs in four amino acids from the H3K36 peptide^19^: A31R, T32F, K37R and H39N. **c** Examples of extended solution structures of ssK36 and H3K36 observed in the MD simulations. The H3K36 peptide is depicted in cyan, the residues altered in ssK36 are coloured blue. The designed ssK36 is shown in black and the ssK36 specific residues in orange. For both peptides, the target lysine 36 in coloured pink.

The substrate specificity of SETD2 for H3K36 has been previously mapped, revealing that the “natural” H3 residues were not the most preferred ones at many positions of the target sequence^19^. Based on this, an artificial peptide substrate was designed that contained the most favourable residues at each position (Fig. 1). It was shown that this 15 amino-acid long super-substrate peptide (ssK36), which differed at four positions from the original H3K36 (A31R, T32F, K37R and H39N) was methylated more than 100-fold faster than the corresponding natural H3K36 peptide. The crystal structure of the ssK36 peptide complexed to the SET domain of SETD2 was resolved, and subtle differences to the H3K36-SETD2 structure were observed, which hint towards the mechanism of the improved methylation of the ssK36 substrate^19^. The four amino acids altered in ssK36 establish different contacts compared to the canonical H3K36 residues, showing that ssK36-R31 forms an H-bond/salt bridge with SETD2-E1674, ssK36-F32 is bound into a pocket formed by SETD2-E1674 and SETD2-Q1676 and ssK36-R37 interacts with the backbone of SETD2-A1700. However, overall the ssK36- and H3K36-SETD2 complex structures are very similar, with an RMSD of both peptides of only 0.308 Å. Therefore, the crystal structures could not fully explain the huge enhancement in the methylation rate of ssK36.

In this work, a combination of molecular dynamics (MD) simulations and biochemical experiments were applied to identify the mechanisms responsible for the increased activity of SETD2 towards ssK36. Multiple steps of the catalytic cycle were investigated to examine the molecular processes explaining why SETD2 has such a strongly increased activity for the super-substrate peptide ssK36, viz. (a) the peptide conformation in solution, (b) the binding process of the peptide into the peptide binding cleft of SETD2 and (c) the approach of a SETD2-peptide complex towards catalytically competent conformations. Strikingly, we observed that ssK36 preferably adopts a hairpin conformation in solution with the target lysine in the middle of the loop facing outside, whereas the H3K36 peptide preferentially has an extended conformation. We next showed that peptides in a hairpin conformation have easier access to the active site of SETD2 compared to the extended conformation. These simulation results were validated by experimental FRET studies and peptide methylation studies with substrates exhibiting stable hairpin conformations. Moreover, in MD simulations of H3K36- and ssK36-SETD2 complexes, we observed increased numbers of the transition-state (TS)-like structures with ssK36. Our data reveal different dynamic interactions of both peptides with the SETD2 SET domain, which ultimately lead to substrate-specific catalytically competent conformations. Hence, by comparing the natural H3K36 peptide and the designed ssK36 in multiple steps of the catalytic cycle, the molecular mechanisms of the much higher catalytic activity of SETD2 towards ssK36 can be explained by an enhanced rate of association of the ssK36 peptide in a hairpin conformation and in better stabilization of TS-like conformations of the SETD2-ssK36 complex.

## Results

### Investigation of different conformational preferences of unbound H3K36 and ssK36

Our first aim was to find out if the conformation of the designed SETD2 super-substrate peptide ssK36 differs from that of the natural H3K36 peptide substrate. To observe potential conformational differences of both peptides in solution, each peptide was subjected to MD simulations in a water box for a total simulation time of 3.5 µs (50 replicates à 70 ns for each peptide). To generate a variety of random starting positions, each replicate had an independent equilibration phase of 10 ns preceding the following simulation. A total of 350,000 conformers from the MD simulations of H3K36 and ssK36 were processed by the clustering algorithm Enspara^21^ and distributed into clusters based on their conformational similarities measured as backbone atom root mean square deviation (RMSD). Each cluster was represented by a centroid structure, visualizing the peptide conformation, which most accurately characterises the corresponding cluster. To ensure a hypothesis-free clustering and to maximise the sampling space, the trajectories of both peptides were pooled before the clustering. Thereby, each conformation from either peptide had equal chances to be clustered with every other conformation.

**Fig. 2:**
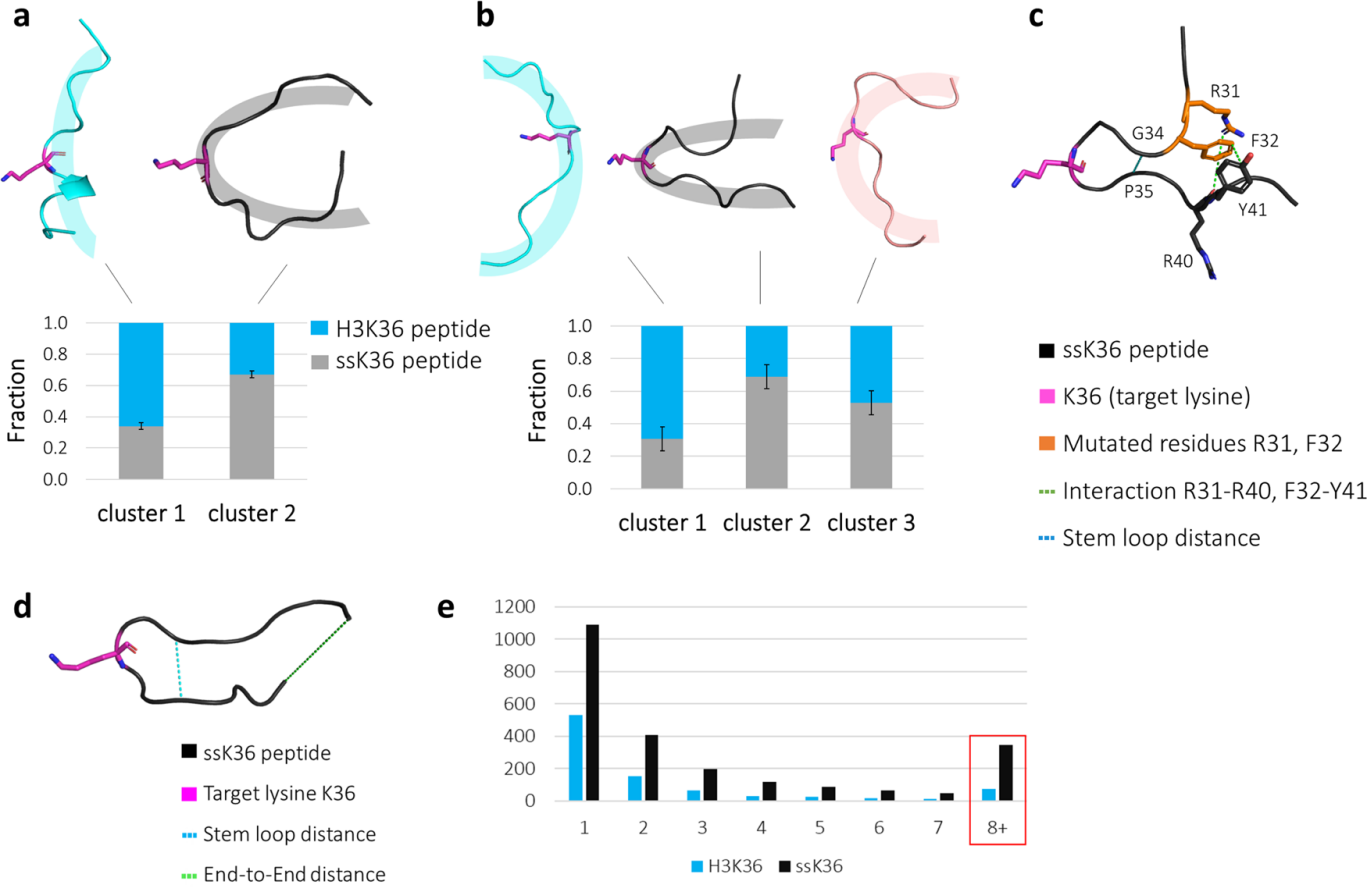
Clustering of the H3K36 and ssK36 peptide solution conformations observed in MD simulations reveals a hairpin conformation preference for ssK36. A total of 350,000 conformations from MD simulations of H3K36 and ssK36 were clustered by Enspara^21^ based on backbone atom RMSD into 2 (**a**) or 3 (**b**) groups. Because of trajectory pooling prior to clustering, the combined conformational space of both peptides was accessible for clustering. Centroid structures of cluster 1 and 2 show the conformation, which most accurately represents the corresponding cluster. **a** Clustering the conformations in two groups revealed major differences between H3K36 and ssK36. Cluster 1 is represented by an almost extended peptide conformation, which consists to 66% of conformations observed in simulations with H3K36. Cluster 2 is represented by a bent hairpin structure, with 67% of the conformations coming from ssK36 simulations. **b** By using three groups for clustering, the different conformational preferences of H3K36 were even more distinct. The extended structure consists to 70% of conformations from H3K36 simulations, whereas the sharply bound hairpin conformation to 69% of conformations observed in ssK36 simulations. Cluster 3 is represented by a mixture of an extended and bent centroid structure and shows an almost equal contribution of H3K36 and ssK36 conformations (H3K36: 47%, ssK36: 53%). The bars represent the mean of three independent clustering replicates. Error bars represent the standard error of the mean (SEM). **c** The ssK36 specific residues F32F and R31 form contacts with Y41 and R40 supporting the formation of a hairpin conformation. Shown is the ssK36 peptide in hairpin conformation taken from one MD simulation with the target lysine, the two specific residues and their interactions partners represented as sticks. **d** For further analysis, structures were assigned as hairpin conformations based on two parameters, a stem loop distance (G33 Cα–P38 Cα) < 7 Å and an end-to-end distance (A29 Cα–P43 Cα) < 15 Å. **e** Distribution of the lifetimes of hairpin conformations, determined by the number of consecutive frames with the peptide in hairpin conformation in all peptide in solution simulations (50 × 70 ns for each peptide). Note the strong overrepresentation of long-living hairpin conformations of ssK36 (highlighted by the red box).

Clustering into 2 groups revealed one cluster with an extended centroid structure and one with a hairpin-like bent conformation (Fig. 2a). Increasing the number of clusters led to three distinct centroid structures: one almost extended (similar to the one observed after clustering into two groups), one sharply bent with strong curvature centred at the target lysine residue and one mixture between the extended and hairpin conformation (Fig. 2b). The largest conformational differences between the centroid structures were observed between cluster 1 (extended) and cluster 2 (bent) (see Supplementary Table 1). Further increasing the number of clusters led to a splitting of the mixed clusters into sub-clusters, while the general extended and bent centroid structures for clusters 1 and 2 were maintained (Supplementary Fig. 1A). Repeating the clustering analysis with randomly selected subsets of the data (50%, 30% and 10%) led to similar centroid structures and comparable distributions of H3K36 and ssK36 among them indicating that the simulation time was sufficient to draw these conclusions (Supplementary Fig. 1B–E).

Next, it was determined how many conformations of the H3K36 or ssK36 simulations were assigned to the respective clusters. This analysis revealed that cluster 1 mainly consisted of conformations observed in simulations of the H3K36 peptide, while the majority of conformations in cluster 2 were produced in simulations with ssK36. Cluster 3 showed an almost equal contribution of H3K36 and ssK36 conformations. This distribution suggests that the H3K36 peptide preferably adopts an extended conformation, whereas the ssK36 peptide prefers a hairpin conformation with a loop at the position of the target lysine. To assess the statistical significance of the 2.2-fold hairpin preference of ssK36, clustering was conducted in three independent runs, which were analysed separately. Based on this, the preference of H3K36 to adopt an extended conformation and ssK36 to adopt a hairpin-like bent conformation was significant. *t*-test analysis of the results of the three independent MD analyses revealed *p*-values of 6.4 × 10^−5^ for the enrichment of H3K36 and ssK36 in clusters 1 and 2 in the two-state clustering (based on two-tailed *t*-test with equal variance). In the three-state clustering, *p*-values of 0.0036 and 0.0032 were obtained for the enrichment of H3K36 in cluster 1 and ssK36 in cluster 2. Additionally, we analysed the frequency of long-lasting hairpin conformations in the MD simulations of both peptides, defined as multiple consecutive MD simulation frames which are in hairpin conformation (Fig. 2d, e). This analysis revealed that ssK36 displayed 4.8-fold more hairpin conformations with long lifetimes compared to H3K36, again indicating that hairpin stabilization is stronger for ssK36.

A detailed analysis of the bent structures showed that the mutated amino acids in ssK36 are engaged in different intramolecular interactions, which stabilize the hairpin structure (Supplementary Fig. 2A–E). These interactions, among others, include hydrogen bonds of R31 with the backbone atoms of R40 and stacking of F32 and Y41 (Fig. 2c). This indicates that the different conformational preferences of H3K36 and ssK36 directly result from the four amino-acid exchanges in the ssK36 peptide (Supplementary Movie 1).

### Experimental investigation of conformational preferences of the H3K36 and ssK36 peptides

**Fig. 3:**
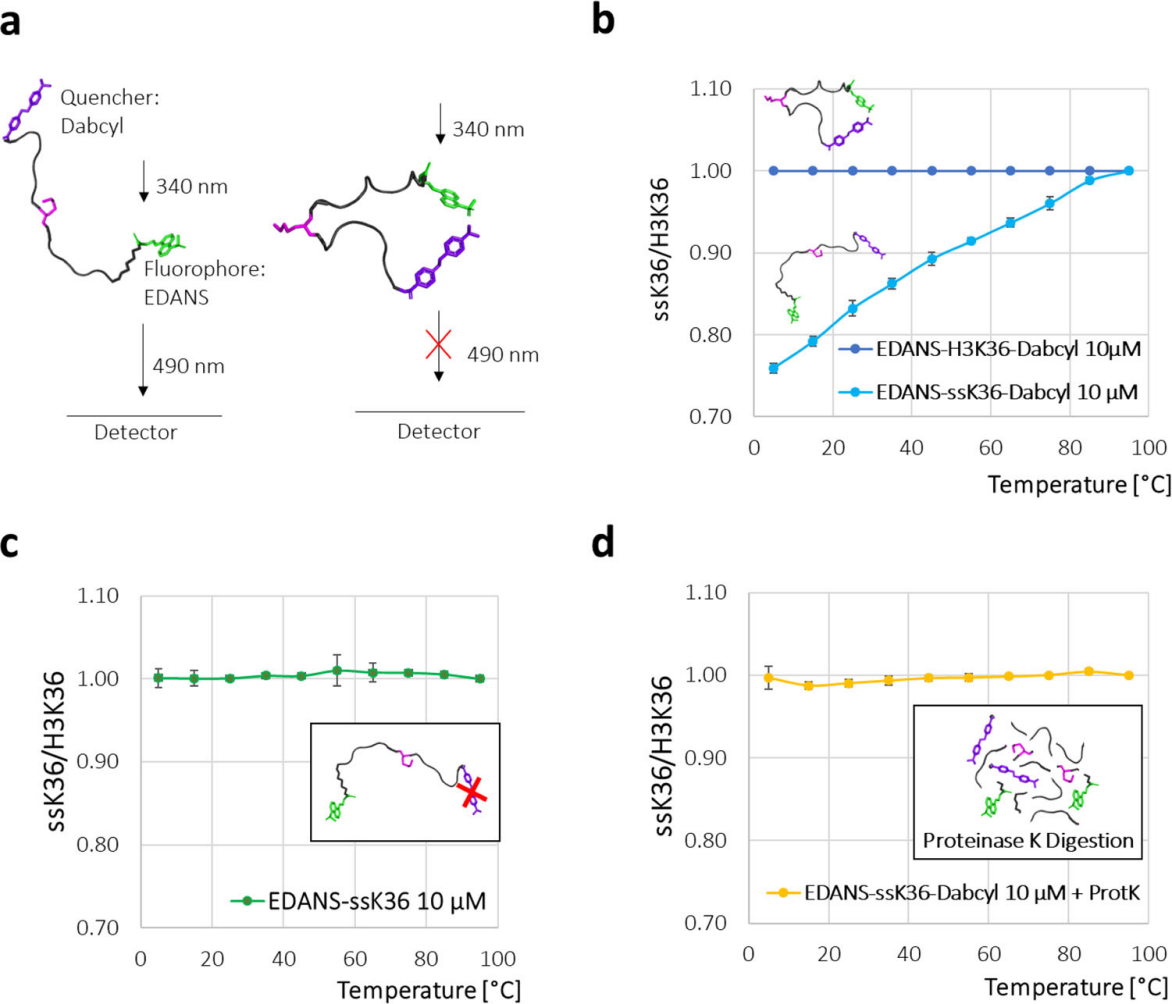
H3K36 and ssK36 peptides show different conformational preferences in solution. **a** Scheme of the FRET system. H3K36 and ssK36 peptides were synthesised with an EDANS fluorophore attached to the C-terminus and a Dabcyl quencher at the N-terminus. EDANS was excited at 340 nm and fluorescence emission was measured at 490 nm. Because of FRET, the fluorescence emission is partially quenched by Dabcyl, and only the remaining fluorescence was measured. The FRET experiments were conducted at multiple temperatures starting at 5 °C up to 95 °C using peptide concentrations of 10 µM. **b** At 5 °C, EDANS-H3K36-Dabcyl showed a 33% higher fluorescence intensity than EDANS-ssK36-Dabcyl. At 95 °C, both peptides displayed the same intensity (corresponding raw data are shown in Supplementary Fig. 3A). **c** Control experiment without quencher showed no temperature-dependent difference between EDANS-H3K36 and EDANS-ssK36 fluorescence. **d** Control experiment showed no difference in fluorescence intensity of EDANS-H3K36-Dabcyl and EDANS-ssK36-Dabcyl after their digestion with Proteinase K. In **b**–**d**, the fluorescence intensity of the ssK36 sample was normalised to the H3K36 sample. Data show average values of two independent experiments, error bars represent the deviation of the data points from the average.

The peptide simulations in solution revealed different conformational preferences for the H3K36 and ssK36 peptides. To challenge these simulation results experimentally, a FRET system was applied to determine the dynamic end-to-end distance distributions of both peptides in solution. For this, an EDANS fluorophore was attached to the C-terminus of the peptides and a Dabcyl quencher to the N-terminus (Dabcyl-peptide-EDANS). EDANS is excited at 340 nm and emits fluorescence at 490 nm. Due to FRET, the emission is partially quenched by Dabcyl depending on the average end-to-end distance and the Förster radius (R_0_) of the FRET pair. Hence, if fluorophore and quencher are in close proximity, more FRET occurs, and less fluorescent light is detected (Fig. 3a). The R_0_ of the EDANS/Dabcyl pair of fluorophores is 33 Å^22^, which is slightly longer than the average end-to-end distance of the peptides in the extended conformation of 27 Å. Hence, even for peptides in an extended conformation, FRET is expected to occur. To assess the dynamic structural properties of both peptides in the FRET experiments, the fluorescence intensity of both peptides was measured at multiple temperatures. Assuming a simple two-state equilibrium between the unfolded and hairpin peptide structures (U-H), the ΔH°’ of hairpin formation should be negative, because of more intramolecular interactions formed in the hairpin state. At the same time, ΔS°’ is expected to be negative as well, because of the reduced conformational freedom of the hairpin conformation. Ignoring the temperature dependence of both terms, with increasing temperature, the unfavourable TΔS term should cause a gradual shift of the equilibrium towards the unfolded conformation.

As shown in Fig. 3b, the intensity of Dabcyl-H3K36-EDANS was 33% higher than the intensity of Dabcyl-ssK36-EDANS at 5 °C. This difference suggests that the ssK36 peptide has a shorter average end-to-end distance. This result is in accordance with a preferential hairpin formation, which brings the quencher and fluorophore closer together, resulting in more FRET and less detectable fluorescence. As expected, the difference between H3K36 and ssK36 decreased with increasing temperature and completely disappeared at 95 °C indicating that the hairpin conformations have unfolded and no conformational differences of both peptides exist at this temperature due to the enhanced thermal movement. After normalisation to this endpoint, the differences between the two peptides were highly reproducible in two independent replicates of this experiment.

To test these observations and tackle the question, of whether the experimental data do indeed show the suggested different conformational preferences, two control systems were used. In one system, only the fluorophore but no quencher was attached to each peptide (Fig. 3c). Both peptides showed a 20-fold higher fluorescence due to the absence of FRET (Supplementary Fig. 3B–D). Moreover, the observed data showed no temperature-dependent differences in their fluorescence. This indicates that the effect, which was seen before, indeed is due to temperature-dependent conformational changes influencing the FRET efficiency.

In a second control experiment, the FRET peptides with quencher and fluorophore were used, but digested with Proteinase K before the measurement (Fig. 3d, for digestion control see Supplementary Fig. 4B). In this system, no conformational preferences are possible anymore. Again, a 12-fold higher overall fluorescence was observed, and no temperature-dependent differences between H3K36 and ssK36 were detected. This result confirms that the intact conformation of the peptides is necessary to observe the temperature-dependent FRET effects.

### Simulation of the peptide association process

**Fig. 4:**
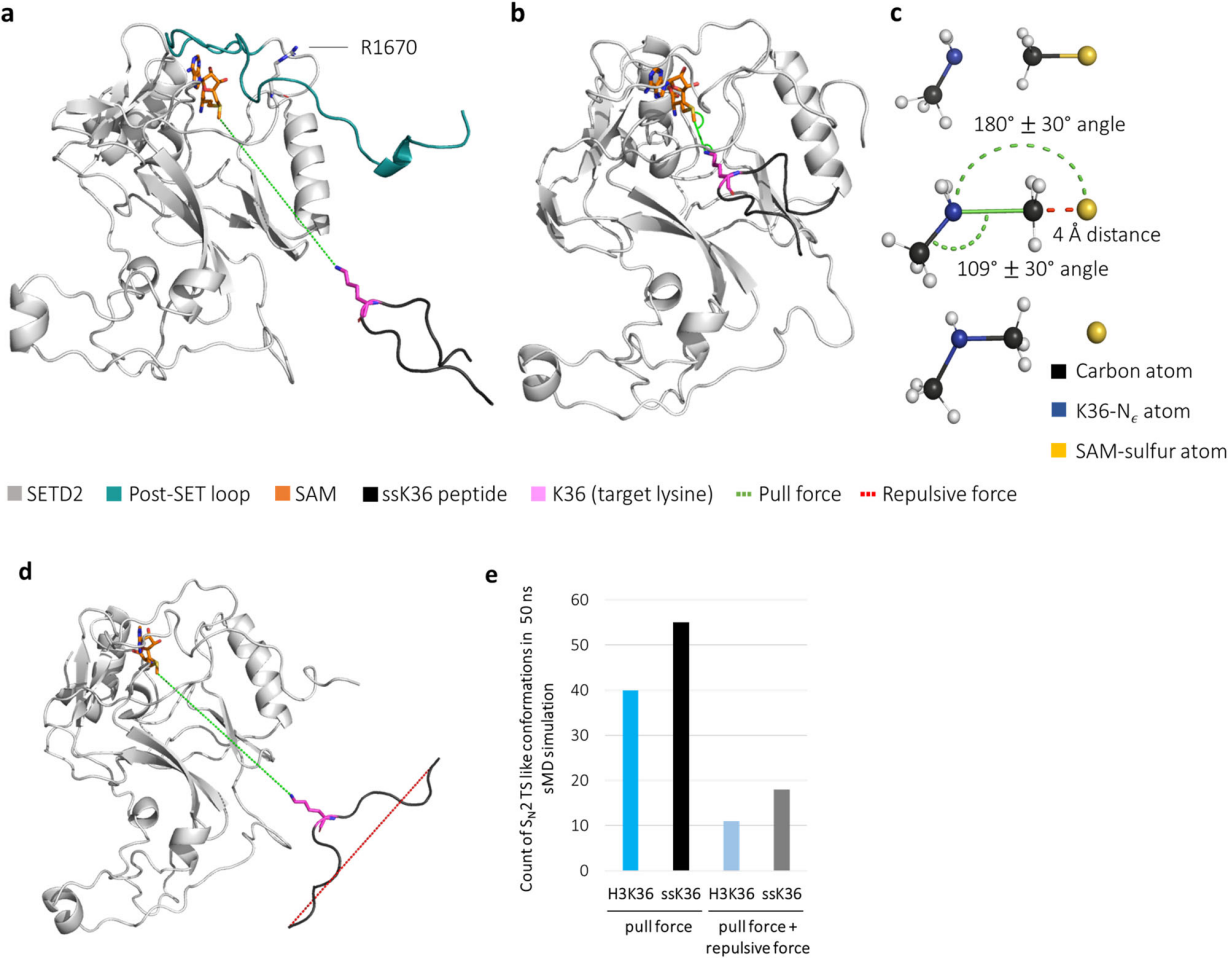
Hairpin conformations facilitate the access of the peptide into the binding cleft of SETD2. **a** sMD simulation of H3K36 and ssK36 driven by a distant dependent external force of 0.5 kJ/(mol × Å^2^) between the Nε-atom of K36 and the methyl group C-atom of SAM. Shown is the starting position of sMD simulation replicates, in which the peptide was positioned 30 Å away from SAM, right above the peptide binding cleft of SETD2 in an open conformation. **b** Example of a successful docking of ssK36 into the active site of SETD2 after 50 ns sMD. **c** Criteria used for definition of a successful docking event derived from the S_N_2 TS-like conformation. **d** sMD simulation with an additional, distance-dependent repulsive force of 0.3 kJ/(mol × Å^2^) between the peptide ends to counteract hairpin formation. **e** Number of successful docking events in 100 sMD simulations with H3K36 or ssK36 based on the TS-like criteria.

The observations in the peptide MD simulations and the solution FRET experiments suggested different conformational preferences for H3K36 and ssK36. To test whether these different conformations of both peptides influence their binding rate to SETD2, steered molecular dynamics (sMD) simulations were applied. sMD simulations use external forces in MD simulations to guide association processes. This accelerates processes that otherwise would be too slow to be modelled and concentrates the sampling along a specific, predefined reaction coordinate^23^. To be suitable for the sMD experiments, SETD2 was modelled in an open conformation, in which no peptide is bound, and the autoinhibitory post-SET loop (Q1676-K1703) is in a detached, open position. In this conformation, the placeholder residue R1670 points outwards, and it does not block the active site^17^ (Fig. 4a). Hence, this conformation represents a state in which a peptide substrate is able to dock into the binding cleft (Supplementary Fig. 5). During the sMD simulations, the loop was not anchored in the open conformation and simulations of the apoenzyme indicated that the loop falls back at timescales >500 ns without peptide. Hence the movement of the loop was slower than the association of the peptide in the sMD experiments, which prevented direct effects of spontaneous loop closure on the sMD simulations. Interestingly, loop closure was observed at shorter timescales after the peptide entered the binding cleft.

For sMD experiments, the H3K36 and ssK36 peptides were placed right above the open binding cleft, 30 Å away from the cofactor SAM with the K36 sidechain facing towards the SETD2 binding cleft. To accelerate the association process, a weak attractive force of 0.5 kJ/(mol × Å^2^) was applied between the N_ε_-atom of lysine 36 and the methyl group C-atom of the SAM (Fig. 4b). In order to define criteria describing a successful docking, the following geometric requirements for a transition-state (TS)-like conformation were derived from the S_N_2 geometry:

1. the distance between the lysine N_ε_ and SAM methyl group C-atom is <4 Å.
2. the angle between the lysine N_ε_ - lysine C_δ_ bond and the forming bond between lysine N_ε_ and the SAM methyl group C-atom is in a 109° ± 30° range.
3. the angle between the lysine N_ε_-SAM methyl group C-atom and SAM methyl group C-atom-SAM S-atom bonds is in a range of 180° ± 30° (Fig. 4c).

One hundred sMD simulations of 50 ns were performed for each peptide, and the number of docking events monitored which fulfilled the success criteria in at least one snapshot. The analysis of the sMD simulations revealed that both peptides were able to establish S_N_2 TS-like conformations following this definition. The H3K36 peptide successfully docked into the active site in 40 of 100 simulations, whereas the ssK36 peptide docked successfully in 55 out of 100 simulations (Fig. 4e). An example of a successful docking event is provided in Supplementary Movie 2.

However, we identified a caveat in this experimental approach. Since lysine 36 is positioned in the middle of the peptide, movement of the peptide caused by pulling with the sMD force on this residue automatically induced the adoption of a hairpin conformation, because the K36 will move towards the SAM while the N- and C-termini of the peptide lag behind. Therefore, both peptides eventually formed hairpin conformations when approaching the active site of SETD2 (90% of H3K36 simulation replicates and 100% of ssK36 simulation replicates at least transiently showed a peptide end-to-end distance <15 Å, Supplementary Fig. 6). To preserve the different conformational preferences of the peptides during the sMD experiment, an additional repulsive force of 0.3 kJ/(mol × Å^2^) between the ends of the peptides was added to the system. This repulsive force increased with decreasing distance between N- and C-termini (N-C distance) and thereby counteracted the formation of a hairpin conformation during the association process (Fig. 4d, Supplementary Movie 3). With this approach, a direct comparison between the docking efficiency of the hairpin and the extended conformations was possible. 100 replicates of this sMD experiment showed that the number of successful dockings strongly decreased if the peptides were not able to approach SETD2 in a hairpin conformation (Fig. 4e). Overall, with the repulsive force, H3K36 docked only 11 times successfully and ssK36 18 times, corresponding to a 3.6-fold (H3K36) and 2.8-fold (ssK36) decrease in docking efficiency when compared to the unconstrained settings. This indicates that the hairpin formation heavily increased the chances of both peptides to successfully dock into the peptide binding cleft of SETD2. Moreover, in both settings, a higher number of productive docking events was observed with ssK36 than with H3K36. All the differences between ssK36 and H3K36 with and without repulsive force are highly significant (Supplementary Fig. 7). This finding underscores the higher intrinsic ability of the ssK36 peptide to adopt a hairpin conformation that is more productive in the association reaction.

### Unfolding of hairpin conformation upon peptide binding to SETD2

**Fig. 5:**
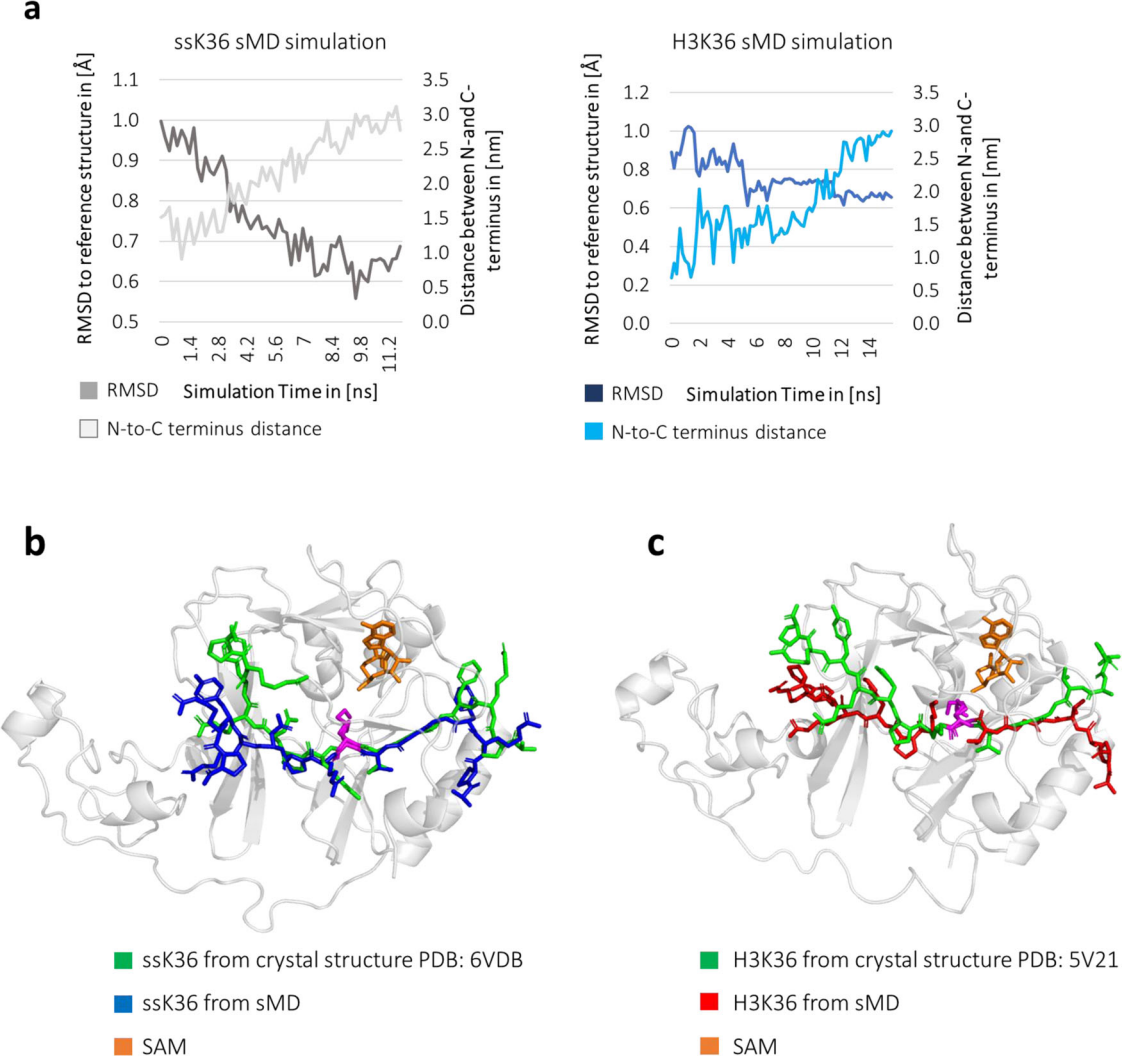
The H3K36 and ssK36 peptides unfold upon binding into the active site of SETD2. **a** RMSD between the peptide conformation in the MD simulation and the extended peptide conformation in the crystal structure (dark grey and dark blue lines) and end-to-end distance of the peptides (light grey and light blue lines) in the sMD simulations without repulsive force which resulted in a TS-like structure. The RMSD was calculated using the crystal structures of complexed peptides in SETD2 as references for the extended conformation after positional optimisation by rigid body movement (PDB: 5V21 for H3K36, PDB: 6VDB for ssK36). The N-C distance was calculated as the distance between the backbone nitrogen atoms of A29 and P43. The RMSD and N-C distance were tracked until the minimum RMSD position was reached. Shown are representative replicates from H3K36 and ssK36 sMD simulations. Additional examples are shown in Supplementary Fig. 8. **b**, **c** Representative structures from sMD simulations with an RMSD of ~0.5 Å overlayed with crystal structures (PDB 6VDB or 5V21).

The sMD experiments showed easier access of hairpin conformations into the active site of SETD2 compared to extended peptide conformations. However, crystal structures of SETD2 complexed with either H3K36 or ssK36 show both peptides bound in an extended conformation in the binding cleft in the SET domain (PDB: 5V21 for H3K36, PDB: 6VDB for ssK36)^17,19^. Therefore, the conformational changes during the sMD simulations were analysed in more detail to find out if, after the initial docking of the hairpin peptide into SETD2, transitions occurred between the hairpin and extended conformations. In this analysis, the crystal structures of the H3K36 or ssK36 peptides bound to SETD2 (PDB: 5V21 for H3K36, PDB: 6VDB for ssK36) were used as reference conformations for peptides in the fully extended state. The conformations of peptides observed in sMD simulations without repulsive force that reached a TS-like structure were compared to the reference conformations based on the backbone RMSD after positional optimisation by rigid body movement. In fact, 84% of ssK36 peptides reduced their RMSD to the extended reference conformation during successful docking events, as well as 68% of the H3K36 peptides, indicating that in most cases, the structures of the docked peptides approached the complex structure observed in the crystal structure analyses (Fig. 5a and Supplementary Fig. 8). It is possible that these numbers would further increase with longer simulation times. In many cases, the final structures after the sMD simulation showed a very good superposition with the extended peptide conformations observed in the crystal structure analysis, in particular at the residues surrounding K36 (Fig. 5b, c).

Since the peptides transiently adopted a hairpin conformation in almost all replicates of the sMD simulations (see above), the reduced RMSD between the peptide structures in the simulation and their crystal structure conformation implies an unfolding process, in which the hairpin structure opens up and the N-C distance increases (Fig. 5a and Supplementary Fig. 8). To monitor this process, the N-C distance of the peptides was determined during the sMD simulations leading to successful docking. For ssK36, 82% of the cases showed an increasing N-C distance during the simulation, as well as 85% of the H3K36 replicates. These analyses indicate that in many simulations during the docking process, both peptides unfold from a hairpin conformation into an extended conformation similar to the crystal structures (an example of this process is provided in Supplementary Movie 4). As expected, the RMSD to the extended reference conformation and N-C distance were highly anticorrelated during the simulations. Moreover, the increases in N-C distance were similar for both peptides indicating that if the transition between a hairpin and extended conformation takes place, both peptides behave similarly.

### FRET analysis of peptide conformations during binding to SETD2

**Fig. 6:**
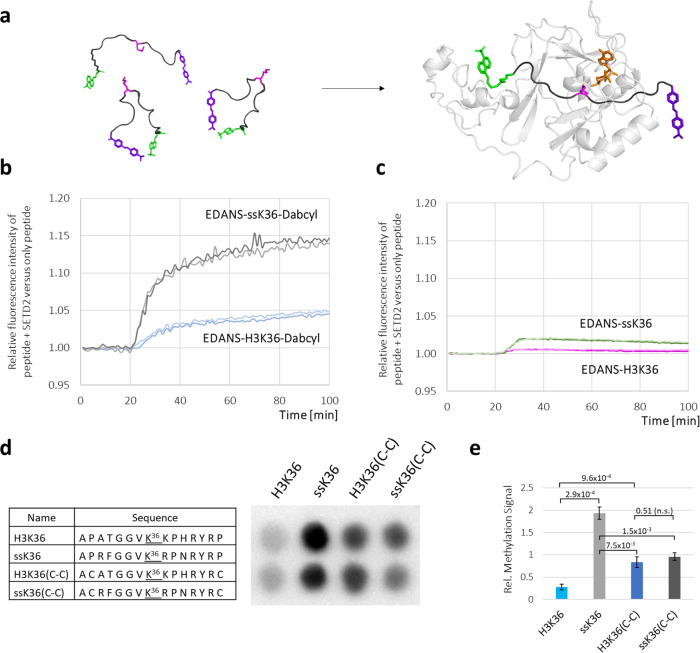
Biochemical analysis of peptide conformation upon binding to SETD2 and peptide methylation experiments. **a** Schematic representation of EDANS-H3K36-Dabcyl and EDANS-ssK36-Dabcyl hairpin conformation unfolding upon binding to SETD2 leading to an increase in the N-C distance and reduced FRET. **b** EDANS-H3K36-Dabcyl (1 µM) and EDANS-ssK36-Dabcyl (1 µM) were dissolved in buffer and the fluorescence was monitored. After 20 min, SETD2 (2 µM) was added to each peptide resulting in an intensity increase of the fluorescence. To monitor fluorescence changes due to the changes in buffer volume and concentrations, control experiments were conducted with addition of buffer to the peptides instead of SETD2. The fluorescence signal of peptides with addition of SETD2 was expressed relative to the signal with addition of buffer. **c** Peptides without quencher were used as a negative control to ensure that FRET was the reason for the fluorescence intensity increase observed in panel **b**. In each panel, the results of two independent experiments are shown. **d**, **e** SPOT peptide array methylation assay to investigate the effect of an enforced hairpin conformation of peptide substrates on SETD2 activity. H3K36 and ssK36 were prepared on a SPOT array together with derivates in which P30 and P43 were altered to C, designated by (C-C). The two Cys residues allow the formation of a disulphide bond that arrests the peptides in the hairpin conformation. The peptide array was then methylated by SETD2 using radioactively labelled SAM and detected by autoradiography. Panel **d** shows one example of an autoradiography image after 2 weeks of film exposure. The two lanes represent technical duplicates and contain identical peptides. Panel **e** shows the average of three independent experiments each containing a technical duplicate set of all spots and the error bars represent the SEM. Signals were normalised to the average signal of all spots in each experiment. *P*-values were determined by *t*-test for a two-tailed distribution of paired values.

The analysis of the sMD simulations suggested that an unfolding of hairpin conformations occurs upon binding of the peptides to the active site of SETD2. To validate this observation experimentally, the H3K36 and ssK36 peptides with attached fluorophore and quencher were used to monitor the peptide conformation by FRET during the binding to SETD2 (Fig. 6a). Strikingly, the addition of SETD2 led to an increase in the fluorescence intensity of both peptides, but the extent of the intensity gain differed (Fig. 6b). The addition of SETD2 to H3K36 caused a 5.0% increase in intensity, while the intensity increased by 14.8% in the case of ssK36. This result suggests that dynamic hairpin conformations of both peptides are resolved during SETD2 binding since fluorophore and quencher are being separated. The finding that the addition of SETD2 caused a three-fold stronger increase in fluorescence of ssK36 than H3K36 can be connected to more hairpin conformations present in solution in the case of ssK36 and/or a more efficient binding of SETD2 to ssK36. To confirm that the intensity increase was caused by the interaction between quencher and fluorophore, peptides without quencher were used (Fig. 6c). Upon addition of SETD2, the intensity increased only slightly for both peptides, much less than for the peptides with the quencher attached (0.18% for EDANS-H3K36 and 1.2% for EDANS-ssK36).

### Methylation experiments with stable hairpin substrates

Our data provide evidence for a previously unknown association pathway of peptides into SETD2, where the binding of a peptide is preferred in a hairpin conformation. During the binding process, the hairpin unfolds, and the peptide adopts the extended conformations seen in the structural studies. To investigate the effect of hairpin formation on the activity of SETD2 directly, we conducted SPOT peptide array methylation experiments with 15mer H3K36 and ssK36 peptides and H3K36(C-C) and ssK36(C-C) variants thereof, which contain two Cys residues at position 30 (P30C) and 43 (P43C) allowing them to form disulphide bonds under oxidative conditions. The disulphide bonds arrest the peptides in the hairpin conformation with K36 presented in the loop of the hairpin. The observed reaction rates (Fig. 6d, e) can be summarized as following:

1. H3K36(C-C) with chemically fixed hairpin conformation is methylated better than H3K36.
2. ssK36(C-C) with chemically fixed hairpin conformation is methylated worse than ssK36.
3. H3K36(C-C) and ssK36(C-C) are methylated almost equally.

H3K36 ≪ H3K36(C-C) ≈ ssK36(C-C) < ssK36

Our finding that the hairpin stabilized H3K36(C-C) is methylated better than the original H3K36 peptide clearly indicates that hairpin formation accelerates the reaction. However, the hairpin peptides H3K36(C-C) and ssK36(C-C) are methylated at a reduced rate when compared with ssK36, indicating that the unfolding of the peptide during complex formation is beneficial for methylation because it allows for the formation of additional contacts between SETD2 and the peptide. Finally, the observation that the hairpin stabilized forms of H3K36 and ssK36 are methylated at similar rates supports the notion that the hairpin formation potential is one of the critical differences between both peptides explaining large parts of their different methylation rates by SETD2.

### MD simulation of the SETD2-peptide complexes

**Fig. 7:**
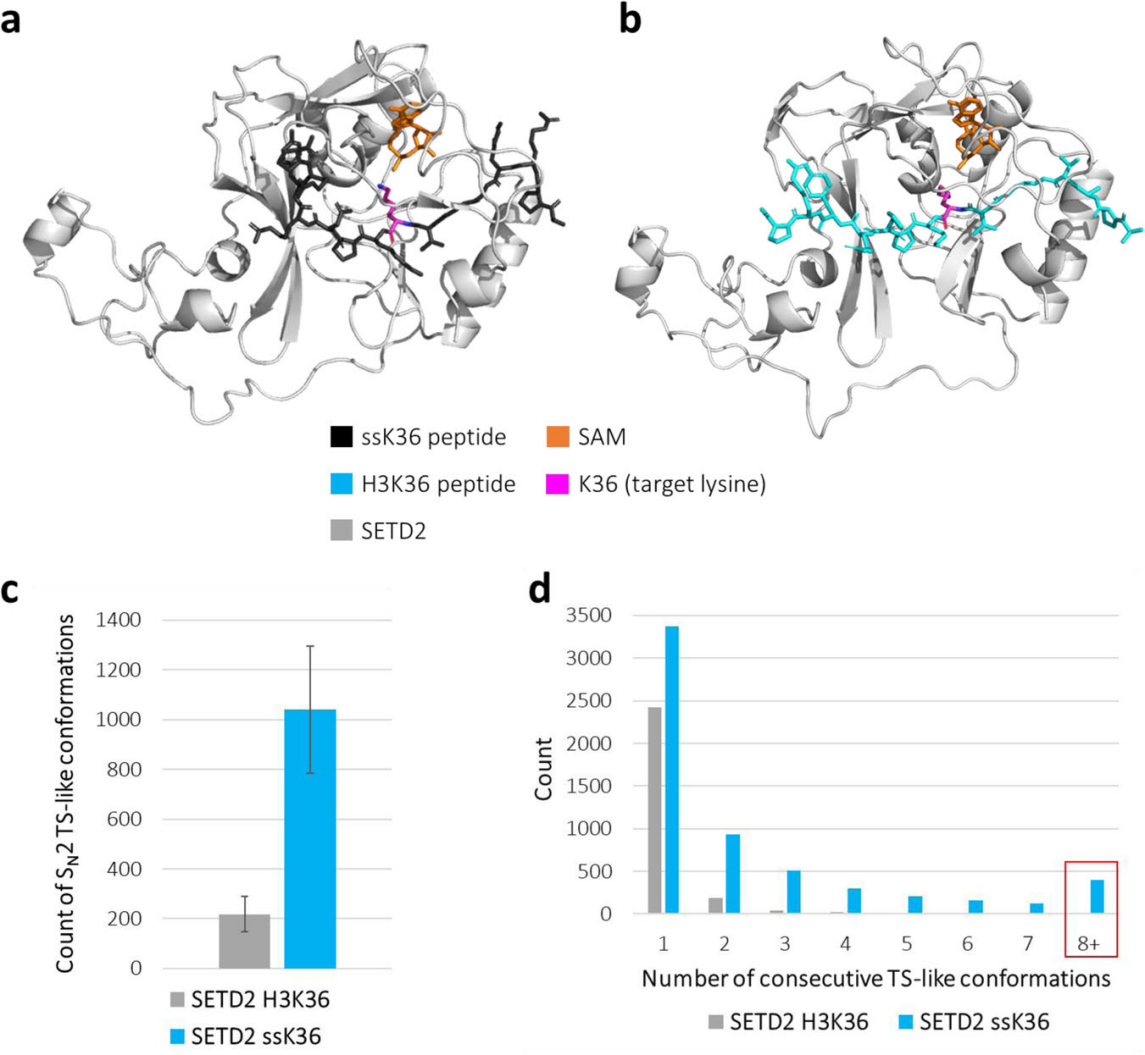
The ssK36 peptide-SETD2 complex establishes significantly more S_N_2 TS-like structures than the H3K36 peptide. **a** Model of SETD2 complexed with ssK36 derived from crystal structure PDB: 6VDB. **b** Model of SETD2 complexed with H3K36 derived from crystal structure PDB: 5V21. **c** Number of TS-like structures reached in MD simulations of the ssK36 and H3K36-SETD2 complexes. The graph shows the average of 15 simulations à 100 ns. Error bars represent the SEM (*p*-value 1.2 × 10^−8^, based on two-tailed *t*-Test with equal variance). **d** Stability of TS-like conformations formed by SETD2-H3K36 and SETD2-ssK36 complexes as determined by the distribution of events with TS-like conformations persisting over a given number of consecutive MD frames. Note the strong overrepresentation of long-living TS-like conformations of ssK36 (highlighted by the red box).

The SETD2 crystal structures with bound ssK36 and H3K36 peptides only resolve the ground state conformation of both complexes. Therefore, we used them as a starting point for MD simulations to investigate the interplay between protein and peptide in atomic detail over time and obtain more information about the dynamic processes leading to enzymatic catalysis (Fig. 7a, b). To derive a parameter that describes the likelihood of catalysis during the MD simulation, the criteria for an S_N_2-reaction TS-like structure as defined above were applied. In a total of 3 µs simulation time (15 simulations à 100 ns for two peptides), ssK36 established significantly more TS-like structures than H3K36. The complex of SETD2 with ssK36 was, on average 1117 times per simulation in a state, in which all TS criteria were fulfilled, and the S_N_2 reaction could take place. In contrast, the complex of SETD2 with H3K36 reached the TS-like conformation only 305 times on average per simulation (*p*-value 1.2 × 10^−8^, based on two-tailed *t*-test with equal variance) (Fig. 7c). Repeating the analysis with randomly selected subsets of the data (50%, 30% and 10%) led to similar ratios between H3K36 and ssK36 (Supplementary Fig. 9).

The increased number of TS-like conformations of ssK36 was accompanied by an increase in the lifetimes of these conformations. As shown in Fig. 7d, TS-like conformations established by H3K36 were mostly transient (only 13 events were identified in which S_N_2 criteria were fulfilled in 8 or more consecutive frames of the simulation). In contrast, the ssK36 peptide achieved long-lasting TS-like states efficiently (397 events fulfilled S_N_2 criteria in 8 or more consecutive frames). Overall, the ssK36 peptide established 30-fold more long-lasting TS-like conformations with SETD2 compared to H3K36.

### Contact analysis of ssK36 and H3K36-SETD2 complexes

**Fig. 8:**
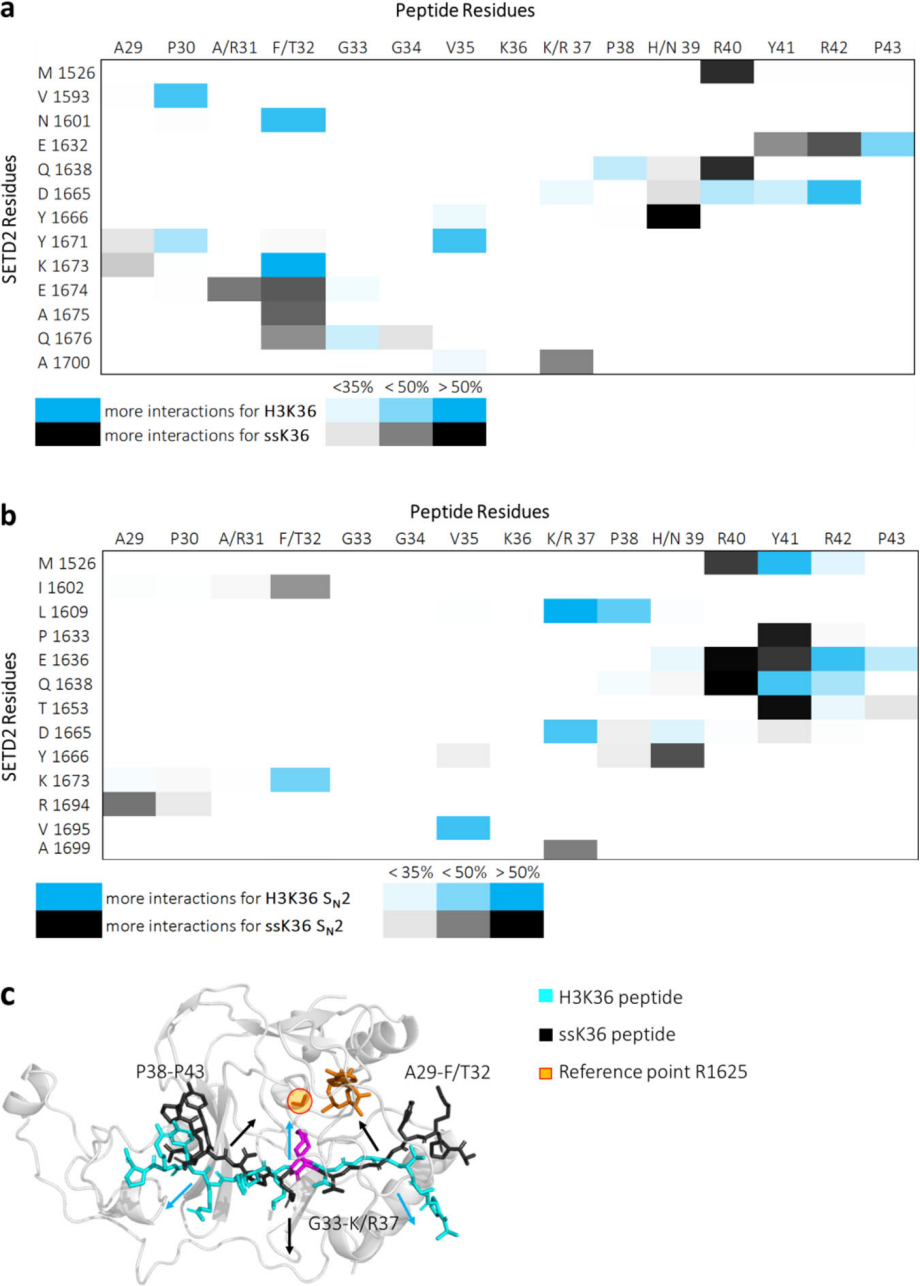
Contact profile*s* of the H3K36 and ssK36 peptides bound to SETD2 observed in the MD simulations. **a** Contact frequency difference (4.5 Å cut-off) of SETD2 simulations complexed with H3K36 or ssK36. Shown are all contacts which exhibited a change over 35% frequency, excluding neighbouring residues. Blue indicates that a specific contact was more often observed in simulations with H3K36. Black symbolizes a higher contact frequency for ssK36. The contacts of A/R31 with E1674, T/F32 with E1674 and Q1676 and K/R37 with A1700 have already been observed in the crystal structure analysis^19^. The contacts of H/N39 with Y1666 and of ssK36-R40 with M1526 and Q1638 have not been described so far. **b** Contact frequency difference of SETD2 simulations complexed with H3K36 or ssK36 in catalytically competent conformations. The T32F mutation caused a different structure in catalytically competent conformations as T32 of H3K36 interacts with K1673, while ssK36-F32 interacts with I1602. Moreover, V35 of H3K36 was engaged in more contacts with V1695 than V35 of ssK36. K37 of H3K36 formed different contacts with L1609 and D1665 compared to ssK36-R37, which was more involved in contacts with A1699. N39 of ssK36 preferably interacted with the backbone atoms of Y1666. R40 of ssK36 was more involved in contacts with M1526, E1636 and Q1638 compared to R40 of H3K36. Y41 of ssK36 interacted with P1633, E1636 and T1653, whereas Y41 of H3K36 interacted with M1526 and Q 1638. Snapshots of interaction differences are shown in Supplementary Fig. 10. **c** Differences in TS-like conformations of H3K36 and ssK36. The distance between the peptide backbone atoms and reference point in the middle of SETD2 was measured. The average distance of N-terminal residues A29–F32 and C-terminal residues P38–P43 of ssK36 to the reference point was lower than for H3K36. However, the residues in the middle of H3K36 were closer to the reference point. See also Supplementary Fig. 11.

To identify the molecular mechanisms leading to the differing abilities of the H3K36- and ssK36-SETD2 complexes to reach TS-like structures, the contacts established between SETD2 and both peptides during the simulations were analysed. The analysis was based on distance criteria, and contacts were considered as established if the distance of a pair of heavy atoms from the peptide and SETD2 was below 4.5 Å. In each case, the fraction of time during which contact was established was analysed to create a contact profile. The resulting contact profiles for H3K36 and ssK36 were then contrasted and differences extracted (Fig. 8a). Previously, three characteristic differences were observed in the static crystal structures of SETD2-H3K36M and SETD2-ssK36M^19^: (1) ssK36-R31 interacts with E1674, (2) ssK36-F32 is placed in a pocket generated by E1674 and Q1676 and (3) ssK36-R37 interacts with the backbone of A1700. These effects were confirmed in the contact profiles observed in the MD simulations (Fig. 8a). Additionally, the contact profile displays a large difference at residue 39, which was not detected in the crystal structure analysis. At this position, H3 carries a histidine (H39) and ssK36 an asparagine (N39). In the ssK36-SETD2 complex, the N_δ_-atom of N39 frequently interacts with the backbone atoms of SETD2 D1665 and Y1666 (Supplementary Fig. 10A). This could cause the backbone atoms of ssK36 N39-P43 to shift and move closer to SETD2 when compared to H3K36. This shift is further supported by the fact that ssK36-R40 is more involved in contact with SETD2, namely M1526 and Q1638, when compared to H3K36-R40 (Supplementary Fig. 10B).

As described above, characteristic differences in the established contacts between H3K36-SETD2 and ssK36-SETD2 were observed in the simulations, which are in good agreement with crystal structure analysis. However, contacts that stabilize catalytically competent conformations able to reach TS-like structures need to be distinguished from the overall contacts. Thus, a more focused view on the catalytically relevant contacts in TS-like conformations was prepared. The resulting contact profile displays where H3K36 and ssK36 bound differently to SETD2 in conformations that reached a TS-like structure. Shown are all contacts, which exhibited a change of over 35%, excluding neighbouring residues (Fig. 8b). This analysis revealed that N39 of ssK36 is engaged in different contacts compared to H39 in H3K36 including the preference of N39 to interact with the backbone atoms of Y1666 as observed before (Fig. 8a). This was also true for R40, as its contact preferences from the previous analysis were also observed in the TS-like conformations. Specific differences in the contact maps of catalytically competent conformations were observed for Y41: Y41 of ssK36 interacts with P1633, E1636 and T1653 of SETD2, whereas Y41 from H3K36 interacts with M1526 and Q1638. As a consequence, the C-terminal part of the ssK36 peptide is shifted towards SETD2 in the catalytically competent conformations when compared with H3K36 (Fig. 8c and Supplementary Fig. 11).

Exemplary snapshots of catalytically competent conformations are shown in Supplementary Fig. 10A–E. The H3K36 peptide shows altered TS-like structures near the target residue for methylation (K36) since V35 of H3K36 has more contact with V1695 than V35 of ssK36. Residue K37 of H3K36 forms different contacts with L1609 and D1665 compared to R37 of ssK36, which is more involved in contact with A1699. The alteration from T to F at position 32 also causes differences in catalytically competent conformations, as T32 of H3K36 interacts with K1673 while F32 of ssK36 interacts with I1602. Because of this, the N-terminal part of ssK36 moves closer to SETD2 in the catalytically competent conformations when compared with H3K36 (Fig. 8c and Supplementary Fig. 11), similarly as observed for the C-terminal part. Strikingly, in catalytically competent conformations the outer parts of the ssK36 peptide, namely residues A29–G33 and P38–P43, approached the reference point inside the protein (R1625), indicating that they come closer to SETD2. However, the middle part showed the opposite trend and slightly detached from SETD2, potentially giving more flexibility for K36 to orient itself (Fig. 8c and Supplementary Fig. 11).

All the peptide interacting residues identified in our work and in the structural analysis^19^ are fully conserved in an alignment of 64 representative mammalian SETD2 homologs (data not shown). We also inspected the literature for data on mutational studies of these residues (Supplementary Table 2)^17,24^. Strikingly, we observed that mutation of peptide interacting residues led to strong reductions in activity if peptide residues conserved between H3K36 and ssK36 are concerned, highlighting the essential roles of these interactions. However, mutations of Y1604 and T1637, which both contact residues in H3K36 that are altered in ssK36, led to an increase in H3K36 methylation, indicating that these regions are not ideal for catalysis in H3K36.

In summary, the different peptide sequences of H3K36 and ssK36 result in different catalytically competent conformations indicating that H3K36 and ssK36 promote catalysis via distinct, separate pathways using different contacts and interactions. According to the results of the MD simulations, the stabilization of catalytically competent conformations in H3K36 is less efficient and leads to fewer catalytically productive binding events. The four mutated residues in ssK36 cause a more efficient formation of catalytically competent conformations, which are different from those of H3K36, by establishing contacts only possible for ssK36, and this finally contributes to a higher enzymatic activity.

## Discussion

PKMTs are essential enzymes, which modify lysine side chains in proteins by the addition of up to three methyl groups. The methylated lysine residues have important signalling roles in histones and non-histone proteins^2,3^. To distinguish different potential methylation substrates, PKMTs form contacts to the amino-acid residues surrounding the target lysine, which establish a specific readout of the amino acids sequence of the target peptide^25^. Here, we approached the mechanisms of the sequence-specific methylation of PKMT substrates using an artificially designed super-substrate peptide of SETD2 as a model system that had been identified before and shown to be methylated about 100-fold faster compared to the natural H3K36 peptide^19^. The solved crystal structures of SETD2 complexed with the H3K36M or ssK36M peptides^17–19^ represented important steps towards the understanding of SETD2’s substrate specificity by illustrating how H3K36M and ssK36M peptides bind into the active site of SETD2 and which contacts are established between SETD2 and the substrate peptides in each complex. However, despite the discovery of peptide-specific contacts, these structures were very similar overall, and they could not easily explain the 100-fold increase in the methylation rate of ssK36. One reason for this is that static crystal structures only capture the conformation of the ground state binding complex, and it is unknown to which extent this represents the catalytically competent conformations able to reach the TS of the enzymatic reaction. Moreover, complex structures do not describe the pathway of the association of the peptide to the enzyme, nor the different structures of the peptides in the solvent, which may affect the association reaction. Hence, critical questions regarding the substrate-specific activity of SETD2 remained unsolved.

Molecular dynamics simulations and protein modelling have the capacity to resolve steps and conformational distributions involved in protein-ligand association reactions and during enzymatic catalysis^26,27^. MD simulations combined with quantum mechanics have been applied to PKMTs to determine details about their catalytic mechanism and product specificities^28–32^. Moreover, MD simulations were applied to investigate conformational changes of PKMTs^8,30,33,34^ or their interaction with SAM and inhibitors^33,35^. Using MD simulations combined with biochemical FRET and peptide methylation experiments, in this study, we aimed to provide a comprehensive view of the role of the substrate peptide sequence in different steps critical for enzymatic turnover, viz. (1) the conformation of the substrate in solution, (2) the pathway of enzyme-substrate association and the conformational changes accompanying it and (3) the approach of the enzyme-substrate complex towards the TS of the methyl group transfer reaction.

**Fig. 9:**
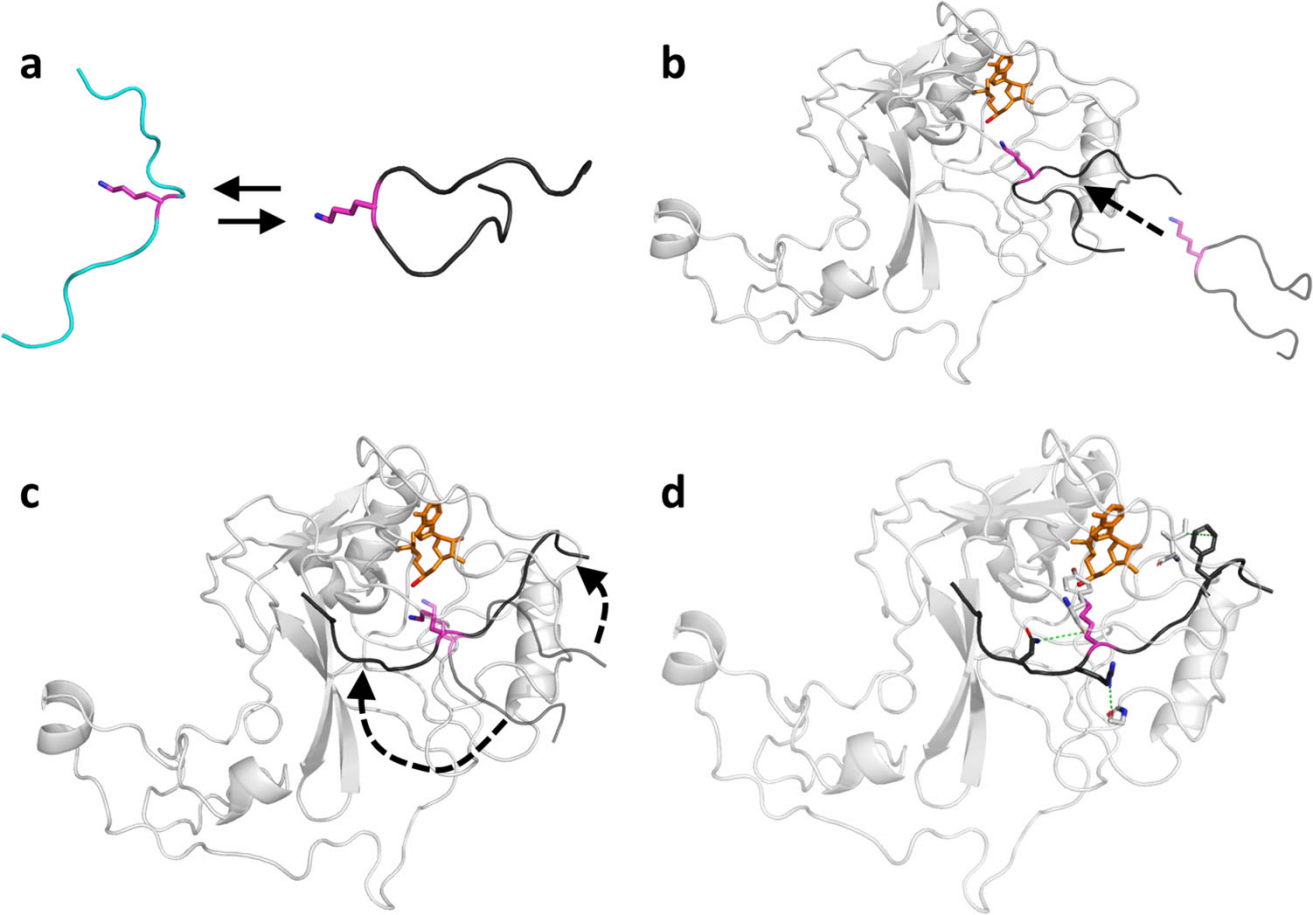
Summary of the results of this study. **a** Preferred hairpin formation of ssK36 in solution due to favourable intrapeptide contacts. **b** Accelerated binding of the hairpin into the SETD2 peptide binding cleft with the K36 facing outside. **c** Unfolding of the hairpin conformation in the SETD2-peptide complex by specific enzyme-peptide contacts in a zipper-like process leading to the adoption of an extended peptide conformation. **d** Distinct contact networks lead to different TS-like conformations and better stabilization of TS-like conformation of ssK36 than H3K36.

Based on our data, the following model of the peptide binding process to SETD2 was developed (Fig. 9): The ssK36 peptide preferentially adopts hairpin conformation in solution, while H3K36 prefers to exist in an extended conformation (Fig. 9a). This difference is a direct result of the four introduced mutations because multiple peptide-specific contacts are established that stabilize the hairpin conformation of ssK36. The hairpin conformation of the ssK36 peptide in solution was confirmed by FRET experiments. Remarkably, the hairpin conformation presents K36 and the adjacent V35 in the loop region facing outside, suggesting that these residues could act as first contact points during the docking of the substrate into the SETD2 SET domain. sMD data indicated that hairpin conformations facilitate the entry of the peptide into the binding cleft of SETD2 when compared with extended peptide conformations suggesting that a transient hairpin conformation is needed during the initial docking of the substrate peptide to SETD2 (Fig. 9b). This result was also confirmed by peptide methylation experiments showing that H3K36 with artificially introduced hairpin conformation is methylated faster. After the formation of the initial SETD2-peptide complex mediated by loop residues V35 and K36, additional contacts between the peptide and SETD2 can then gradually spread towards C- and N-termini of the peptide in a zipper-like process. This leads to an unfolding of the hairpin and adoption of an extended peptide conformation as observed in the crystal structures of SETD2 complexed with either H3K36 or ssK36 (Fig. 9c). We found in the sMD simulations and FRET experiments that the N-C distance of the peptides increased during the peptide association process, when the RMSD of the conformations in the sMD and the crystal structures decreased, suggesting a gradual unfolding of the hairpin conformation for both peptides during the binding process. Preventing peptide unfolding reduced the methylation by SETD2, as seen in the methylation of ssK36 with chemically fixed hairpin conformation. The dynamic peptide association mechanism described in this work complements the understanding of the dynamic behaviour of the post-SET loop of the enzyme during peptide binding^17^.

Mechanistically, the preference for this association pathway may be related to the fact that SETD2 has an extended peptide binding cleft that can change between an open and a closed conformation in a peptide free state. In the hairpin conformation, K36 and V35 have maximum flexibility to position themselves and bind to their respective hydrophobic pockets in the SETD2 active site. Importantly, for this interaction, only the corresponding part of the binding cleft has to be in an open access state. The strong interaction of SETD2 with V35 and K36 could support long-lasting binding events of the peptide after the initial docking. During this phase, the transient opening of further parts of the binding cleft can lead to a stepwise formation of the other peptide-SETD2 contacts starting from the central part of the complex (V35 and K36) in a zipper-like process until the final stable complex is formed. In contrast to this stepwise process, direct binding of the peptide in an extended form would require a completely open binding cleft and this pathway is disfavoured for this reason.

This model is in agreement with several data from the literature: (1) The finding that hydrophobic residues at the V35 position are obligatory for substrate recognition by SETD2^19^. (2) The key role of K36 in the binding of ssK36 to SETD2 is illustrated by the finding that the inhibition constant of the K36M mutant of ssK36 was only moderately (3.5-fold) improved as compared to the K36M mutant of H3K36, while the stimulation of ssK36 methylation was much stronger^19^. These results suggest that the outwards-faced V35 and K36 in the hairpin directly influence the binding process. (3) Previous investigations showed that the SET-domain containing PKMT SET7/9 had an increased methylation activity towards an artificially designed peptide with a disulphide-stabilized hairpin structure^36^. This could indicate that peptides with hairpin conformations, in general, have favourable catalytic properties for PKMT-catalysed peptide methylation.

In the next step of the enzymatic turnover cycle, the results from MD simulations of the SETD2-peptide complexes revealed a specific TS-like conformation for ssK36 that is stabilized more efficiently in SETD2-peptide complexes than the TS-like conformation of H3K36 (Fig. 9d). Our analysis confirmed findings from the previous crystal structure analysis^19^, but important additional contact differences were identified. The analysis of enzyme-peptide contacts in catalytically competent conformations observed in the MD simulations showed that H3K36 and ssK36 form distinct contact networks with the enzyme, indicating that methylation of both peptides proceeds through different TS-like conformations. The four introduced mutations formed unique enzyme contacts exclusively accessible for ssK36. Because of these contacts, the N- and C-terminal parts of ssK36 move closer to SETD2 when compared with H3K36. However, the central part of the peptide shows the opposite trend and slightly detaches from SETD2, potentially giving more flexibility for K36 to re-orient. This analysis raises the striking possibility that methylation of different peptide sequences by one PKMT may generally involve alternative substrate-specific conformations of the catalytically competent enzyme-peptide complex, which widens our understanding of the mechanism of this important group of enzymes.

Based on our data, the roughly 100-fold increase in methylation rate of ssK36 can be attributed to two distinct effects, the accelerated association due to preferred hairpin formation and the better stabilization of TS-like conformations. The finding that kinetic barriers during association and formation of TS-like conformations are lowered with ssK36, is in agreement with previous data showing that the K_M_-value of ssK36 is only marginally improved (1.4-fold), leaving most of the rate enhancement for k_cat_ term (about 70-fold)^19^. The overall rate enhancement of ssK36 is in a very good agreement with the results of the MD simulations presented here because the 4.8-fold increase in the stable hairpin formation of ssK36 combined with the 30-fold increase in TS-like conformations with long lifetimes could explain an up to 140-fold increase in reaction rate. Currently, it is not possible to determine the minimal lifetimes of hairpin conformations and TS-like structures that are needed for productive association and catalysis. Hence, the 140-fold change represents an upper limit of the potential effect, because the differences in both properties are smaller if lifetimes were not considered. The sMD experiments support the proposed pathways of hairpin peptide conformation during the association process followed by the unfolding of the hairpin and positioning of the peptide in the SETD2 binding cleft. While they also qualitatively revealed improved efficiency of ssK36 in the association process, quantitative statements cannot be made, because the sMD approach includes external forces.

We conclude that the mechanistic principles of hairpin association and substrate-specific catalytic conformations discovered here shed new light on the catalytic mechanism of SETD2, an important enzyme in the physiology of human cells. They may apply to other PKMTs as well, thereby helping to understand the principles of catalysis of this large and highly relevant group of chromatin and protein-modifying enzymes with very important roles in cellular signalling. Moreover, the hairpin-based association mechanism may open new avenues for PKMT inhibitor design by using hairpin peptides as design scaffolds.

## Experimental procedures

### General settings and parameters in the molecular dynamics simulations

All molecular dynamics (MD) simulations were performed in OpenMM 7.4.2^37,38^ utilizing the NVIDIA CUDA^39^ GPU platform. The systems were parameterised using the General Amber force field (GAFF) and AMBER 14 all-atom force field^40–42^. SAM was modelled based on the coordinates of SAH and parametrised using ANTECHAMBER from AmberTools (18.0)^43^. The non-bonded interactions were treated with a cut-off at 10 Å. Additionally, the Particle Mesh Ewald method^44^ was used to compute long-range Coulomb interactions with a 10 Å non-bonded cut-off for the direct space interactions. Energy minimisation of the system was performed until a 10 kJ/mole tolerance energy was reached. Simulations were run using a 2 fs integration time step. The Langevin integrator^45^ was used to maintain the system temperature at 300 K with a friction coefficient of 1 ps^−1^. The initial velocities were assigned randomly to each atom using a Maxwell–Boltzmann distribution at 300 K. A cubic water box with a 10 Å padding to the nearest solute atom was filled by water molecules using the tip4p-Ew model^46^. The ionic strength of 0.1 M NaCl was applied, by adding the corresponding number of Na^+^ and Cl^−^ ions. Protonation states, equilibration protocols and other specifications for the individual system setups are described below. Production runs were performed under periodic boundary conditions, and trajectories were written every 10,000 steps (20 ps).

### Simulation of peptides in solution

The peptide structures were retrieved from crystal structures of the complexed peptide with SETD2, PDB: 5V21 for the H3K36 peptide and PDB: 6VDB for the ssK36 peptide (Fig. 1a–c). Total number of atoms: 15753 for H3K36: 13 Cl^−^ ions, 9 Na^+^ ions, volume of the box: 142,713 Å^3^; total number of atoms: 17,236 for ssK36: 15 Cl^−^ ions, 10 Na^+^ ions, volume of the box: 159,179 Å^3^. Missing amino acids were modelled by the PyMOL (2.4.1) builder. Since the available crystal structures of SETD2 are complexed with K36M inhibitor peptides, M36 was mutated to K36 for both peptides using the PyMOL (2.4.1) mutagenesis tool. To equilibrate the solvent, a 5 ns pressure coupled equilibration with Monte Carlo barostat^47^ was performed at a pressure of 1 atm, which was followed by a 10 ns free equilibration without constraints. The equilibration steps were repeated for each simulation replicate to start the 70 ns production simulation with a variety of peptide starting positions.

### Simulation of peptide association to SETD2

The structure of human SETD2 was retrieved from the Protein Data Base (PDB: 6VDB, all residue numbers in this work refer to this entry) and missing amino acids were modelled using PDBFixer^48^. The complexed peptide was deleted from the structure, and the centroid structures from the clustering of peptide conformations in solution, as described in the chapter Simulation Analysis, were placed 30 Å away from the binding cleft (distance between SAM methyl group and sidechain nitrogen of the peptide lysine 36). The target lysine was manually deprotonated as required for the S_N_2 mechanism^7^. The p*K*_a_ values of SETD2 protein were calculated at pH 7 using PROPKA^49,50^ provided by the PDB2PQR server (version 2.0.0)^51^. The p*K*_a_ values of peptide were assigned according to the AMBER ff14SB force field^52^. The Zn^2+^ ions were modelled using the cationic dummy atom method^53–55^. The cysteines 1499, 1501, 1516, 1520, 1529, 1533, 1539, 1631, 1678, 1680, 1685 were treated as unprotonated to ensure proper Zn^2+^ binding^56^. The protein charge was neutralised by adding 7 Cl^−^ ions.

To model SETD2 in an open position, the post-SET loop (Q1691-K1703) was manually lifted upwards using PyMOL (2.4.1)^57^ based on the results described in Yang et al. 2016^15^. In this conformation, the peptide binding cleft is no longer blocked by the post-SET loop and the autoinhibitory residue R1670 points outwards. The post-SET loop was not constrained in the open conformation. Simulations of the apoenzyme indicated that the loop falls back at timescales >500 ns without peptide, which did not interfere with the sMD experiments, which were conducted over 50 ns. However, in the sMD simulation replicates, contacts of the post-SET loop with the docked peptide were also observed to occur at shorter timescales.

The open position of SETD2 was then used for the sMD simulations^23^ (H3K36: total number of atoms 89,040, the volume of box 571,658 Å^3^; ssK36: total number of atoms 98,080, the volume of box 674,494 Å^3^). To equilibrate the solvent, a 5 ns pressure coupled equilibration with Monte Carlo barostat was performed at a pressure of 1 atm. The C-alpha (Cα) atoms of SETD2, the peptide and the SAM atoms were restrained with a force of 100, 100 and 5 kJ/mole × Å^2^, respectively. The restraints were taken off successively, starting with the SETD2 Cα restraints, followed by a 5 ns equilibration with the peptide and SAM still being restrained. Subsequently, the SAM and peptide restraints were removed as well, followed by 0.1 ns equilibration with no restraints. A distant dependent force of 0.5 × distance of centroid 1 (K37 atoms NZ, HZ1, HZ2) and centroid 2 (SAM atoms SD, CE, H10) (kJ/mol)/Å^2^) was used to pull the centre of mass (COM) of the lysine 36 sidechain nitrogen and its attached two hydrogen atoms, towards the COM of the SAM methyl group and its attached three hydrogen atoms. The sMD simulation was run for 100 replicates à 50 ns (total simulation time 5 µs).

To discriminate between the different conformational preferences of the peptides in the sMD experiment, an additional, distance-dependent repulsive force of −0.3 × distance centroid 1 (A29 atoms N, CA, C) and centroid 2 (P43 atoms CG, CD, OXT) (kJ/mol)/Å^2^) was added to the system. This repulsive force decreased with the distance of A29 and P43, and it pushed the peptide ends apart if they got in close proximity, thereby preventing the formation of a hairpin conformation.

### MD simulation of SETD2-peptide complex conformations

The starting structures were retrieved from the Protein Data Base, PDB: 5V21 for SETD2 with the H3K36 peptide, PDB: 6VDB for SETD2 with the ssK36 peptide. Missing amino acids, M to K mutations, preparation of SAM and the Zn^2+^ ions was carried out as described in the previous section (H3K36: total number of atoms 70,956, the volume of the box 461,784 Å^3^; ssK36: total number of atoms 70,292, the volume of the box 440,548Å^3^). A 5 ns pressure coupled equilibration with Monte Carlo barostat was performed at a pressure of 1 atm. SETD2 and peptide C-alpha (Cα) atoms, as well as cofactor SAM atoms, were restrained with a force of 100 and 5 kJ/mole × Å^2^, respectively. The restraints were taken off successively, starting with the Cα restraints, followed by a 5 ns equilibration with only SAM restrained. Subsequently, the SAM restraints were removed as well, followed by 5 ns equilibration with no restraints. For production, 15 replicates à 100 ns were performed (total simulation time 1.5 µs).

### Analysis of the MD simulation data

The data analysis was performed utilizing MDTraj (1.9.4)^58^, Enspara (0.1.1)^21^ and contact-map-explorer (0.7.1)^59^. The clustering of the peptide trajectories with Enspara was carried out using the k-hybrid algorithm based on backbone atom (Cα, C and N) root mean square deviation (RMSD). For the contact-map-explorer, a cut-off of 4.5 Å was used, and the next two neighbouring residues were ignored. A contact was counted if at least one heavy atom of a residue was in a 4.5 Å^3^ sphere of one heavy atom from another residue. All structures were visualized using PyMOL (2.4.1)^57^.

### SETD2 cloning, expression and purification

The His_6_-tagged expression construct of SETD2 catalytic SET domain (amino acids 1347–1711, UniProt No: Q9BYW2) was expressed and purified as described previously^19,60^. In short, the plasmid encoding the His-tagged SETD2 SET domain was transformed into BL21 (DE3) Codon Plus cells and protein expression was induced by 0.2 mM IPTG at 17 °C for 16 h. Protein purification was done by affinity chromatography using Ni-NTA affinity agarose resin (Genaxxon bioscience). After washing the beads, the purified protein was eluted in elution buffer (220 mM imidazole, 30 mM KPI, 500 mM KCl, 0.2 mM DTT, 10% glycerol). Fractions containing SETD2 were pooled and dialysed in dialysis buffer (20 mM HEPES pH 7.2, 200 mM KCl, 0.2 mM DTT, 1 mM EDTA, 10% glycerol) for 3 h at 8 °C. Finally, the protein was aliquoted and flash frozen in liquid nitrogen and stored at – 80 °C. The purity of the protein preparation was analysed by sodium dodecyl sulfate–polyacrylamide gel electrophoresis (SDS-PAGE) using 16% gel stained with colloidal Coomassie brilliant blue (Supplementary Fig. 4A).

### FRET analysis of peptide conformations and SETD2 binding kinetics

For the FRET experiments, the Jasco FP-8300 spectrofluorometer was used. The excitation was set to 340 nm, and the emission was measured at 490 nm. The excitation and emission bandwidths were 5 and 10 nm, respectively. Samples were stirred at 600 rpm. The peptides (Table 1) were obtained from JPT Peptide Technologies GmbH and dissolved in DMSO stock solutions at 5 mM. For the experiments, the peptides were diluted in 20 mM HEPES pH 7.2, 200 mM KCl, 1 mM EDTA and 10% glycerol to a final concentration of 10 µM. For control experiments, the peptides were diluted in 20 mM HEPES pH 7.2, 200 mM KCl and 10% glycerol to a concentration of 11 µM and 1/10 of the volume of a Proteinase K solution (100 µg/ml, dissolved in 20 mM HEPES pH 7, 100 mM NaCl and 10 mM MgCl_2_) was added and the mixture incubated for 1 h at 37 °C. The SETD2 binding kinetics were performed using 1 µM peptide dissolved in 20 mM HEPES pH 7.2, 200 mM KCl, 1 mM EDTA and 10% glycerol supplemented with 2 µM of SETD2 catalytic SET domain. Most measurements were performed at 26 °C, but for the analysis of peptide conformations, a temperature gradient from 5 to 95 °C in 10 °C steps was applied.

**Table 1:** Sequences of the peptides used in the biochemical experiments in this study.

Peptides used in the FRET experiments	
Name	Sequence
H3K36	Dabcyl-A P A T G G V K^36^ K P H R Y R P-Glu(Edans)
ssK36	Dabcyl-A P R F G G V K^36^ R P N R Y R P-Glu(Edans)
noQuencher-H3K36	A P A T G G V K^36^ K P H R Y R P-Glu(Edans)
noQuencher-ssK36	A P R F G G V K^36^ R P N R Y R P-Glu(Edans)

Peptides on SPOT peptide arrays used for the methylation experiments	
Name	Sequence
H3K36	A P A T G G V K^36^ K P H R Y R P
H3K36(C-C)	A C A T G G V K^36^ K P H R Y R C
ssK36	A P R F G G V K^36^ R P N R Y R P
ssK36(C-C)	A C R F G G V K^36^ R P N R Y R C

### Peptide array methylation assay

Methylation of peptide SPOT arrays is a powerful method to assess the specificity of PKMTs^61^. Peptide arrays containing fifteen amino-acid long peptides were synthesized using the SPOT synthesis method with an Autospot peptide array synthesizer (Intavis AG, Köln, Germany), basically as described^19^. Briefly, the SPOT array membranes were pre-incubated in Incubation buffer (20 mM Tris/HCl pH 9, 1.5 mM MgCl_2_) for 5 min on a shaker. Thereafter, the membranes were incubated in methylation buffer supplemented with radioactively labelled SAM (PerkinElmer) and 1 μM SETD2 enzyme for 60 min on a shaker. Afterwards, the membranes were washed five times for 5 min in wash buffer (100 mM NH_4_HCO_3_, 1% SDS) and incubated in amplified NAMP100V (GE Healthcare) for 5 min. This was followed by exposure of the membranes to a HyperfilmTM high-performance autoradiography film (GE Healthcare) at −80 °C in the dark. The films were developed with an Optimax Typ TR machine after different exposure times. For quantification, the signal intensities were measured with the ImageJ software and analysed with Microsoft Excel.

### Statistics

*t*-tests were conducted with Excel using the specified settings. *P*-values based on binomial distributions were calculated with Excel using the Binom.dist function.

### Reporting summary

Further information on research design is available in the Nature Research Reporting Summary linked to this article.

## Supplementary information

Jeltsch_PR File

Supplementary information

Reporting Summary

## Data Availability

All biochemical data generated or analysed during this study are included in the published article and its supplementary files. Source data underlying plots shown in the figures and Supplementary Movies 1–4 are provided on DaRUS (10.18419/darus-2508). All other data or sources are available from the corresponding author on reasonable request.

## Code availability

MD simulations codes and analysis scripts are provided on DaRUS (10.18419/darus-2508).
