# Supplementary material for: Mechanistic basis of the increased methylation activity of the SETD2 protein lysine methyltransferase towards a designed super-substrate peptide
Source: Commun Chem. 2022 Oct 28;5:139. doi: 10.1038/s42004-022-00753-w (PMC9814698; doi:10.1038/s42004-022-00753-w)
Supplement: Supplementary file 2 — Supplementary information [file 42004_2022_753_MOESM2_ESM.pdf]

Mechanistic basis of the increased methylation activity of the SETD2 protein lysine methyltransferase towards a designed super-substrate peptide

Philipp Schnee, Michel Choudalakis, Sara Weirich, Mina S. Khella, Henrique Carvalho, Jürgen Pleiss & Albert Jeltsch

Supplementary information

Supplementary Figures

Supplementary Figure 1: Additional data related to the clustering of the H3K36 and ssK36 peptide conformations observed in the MD simulations.
Supplementary Figure 2: Contact profiles of the H3K36 and ssK36 peptides display multiple conformational differences in solution.
Supplementary Figure 3: Additional data related to the FRET experiments to analyse peptide conformations in solution.
Supplementary Figure 4: SDS-PAGE of purified SETD2 enzyme and tricine gel of Proteinase K digested H3K36 and ssK36 peptides.
Supplementary Figure 5: Comparison of SETD2 structures with R1670 turned inwards and outwards.
Supplementary Figure 6: Observation of peptides in hairpin conformation in the sMD experiments.
Supplementary Figure 7: Number of successful docking events in the sMD simulations and corresponding pairwise p-values.
Supplementary Figure 8: H3K36 and ssK36 peptides unfold upon binding into the active site of SETD2.
Supplementary Figure 9: Additional data related to MD simulations of peptide-SETD2 complexes.
Supplementary Figure 10: Visualization of the contact profiles of H3K36 and ssK36 peptides bound to SETD2 observed in the MD simulations
Supplementary Figure 11: Distance difference between the H3K36 and ssK36 peptide nitrogen backbone atoms and a reference point in the middle of SETD2 (R1625).

Supplementary Tables

Supplementary Table 1: All atom RMSD comparison of centroid structures.
Supplementary Table 2: Results of mutational studies of SETD2 residues involved in H3K36 and ssK36 peptide interaction.

Supplementary References

Supplementary Figures

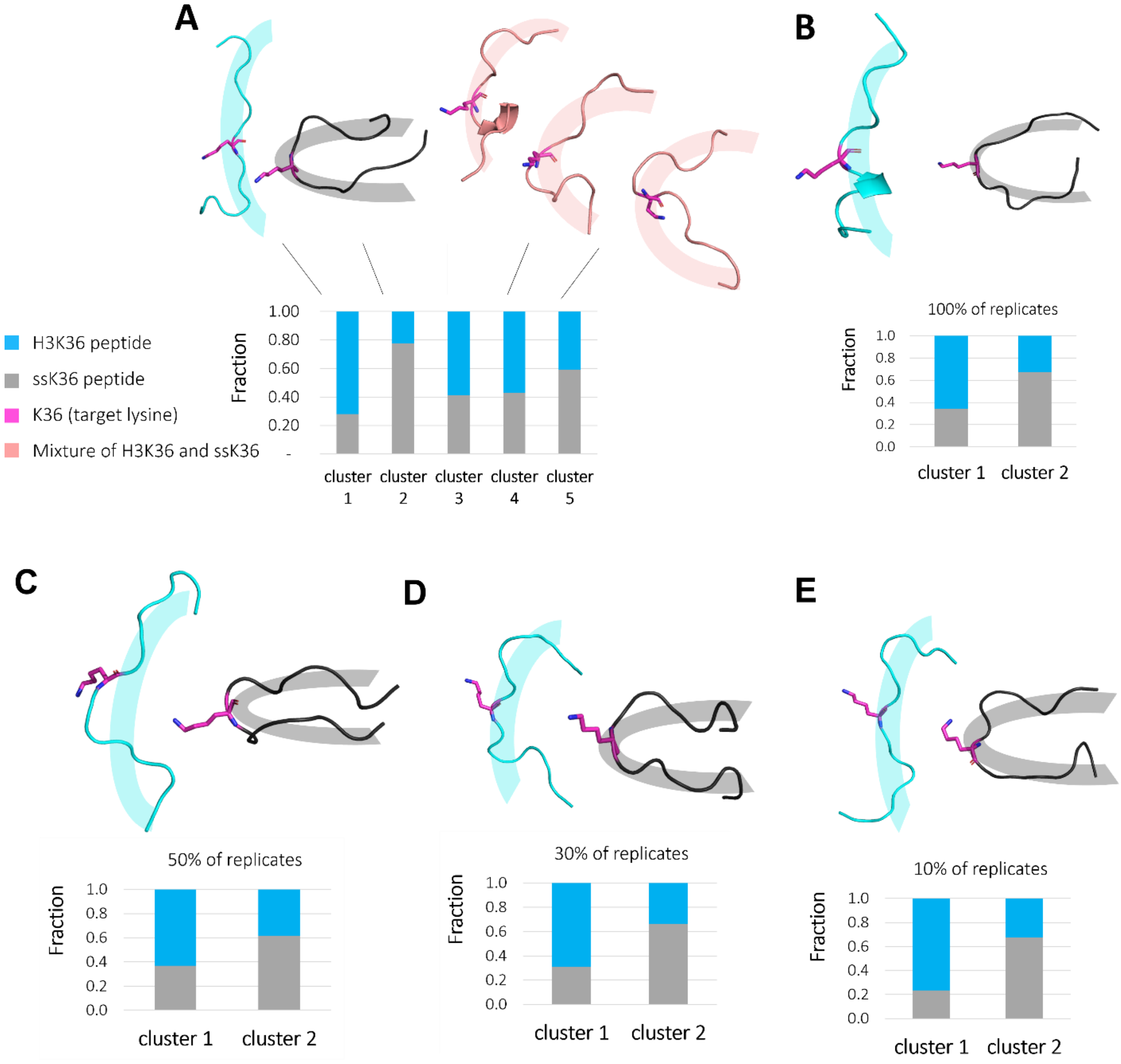

Supplementary Figure 1: Additional data related to the clustering of the H3K36 and ssK36 peptide conformations observed in the MD simulations. A| Clustering of the H3K36 and ssK36 peptide conformations by enspara into 5 groups. The clustering was conducted based on backbone atom RMSD (Cα, C, N). The centroid structures of cluster 1 to 5 with corresponding fractional contribution of conformations from MD simulations with the H3K36 or ssK36 are shown. **B-D|** Subsets of replicates (100% = 50 replicates, 50% = 25 replicates, 30% = 15 replicates and 10% = 5 replicates) were randomly selected and the conformations were clustered in 2 groups. Reducing the number of replicates did not lead to noticeable changes of the centroid structures and distributions of H3K36 and ssK36 conformations between the conformations.

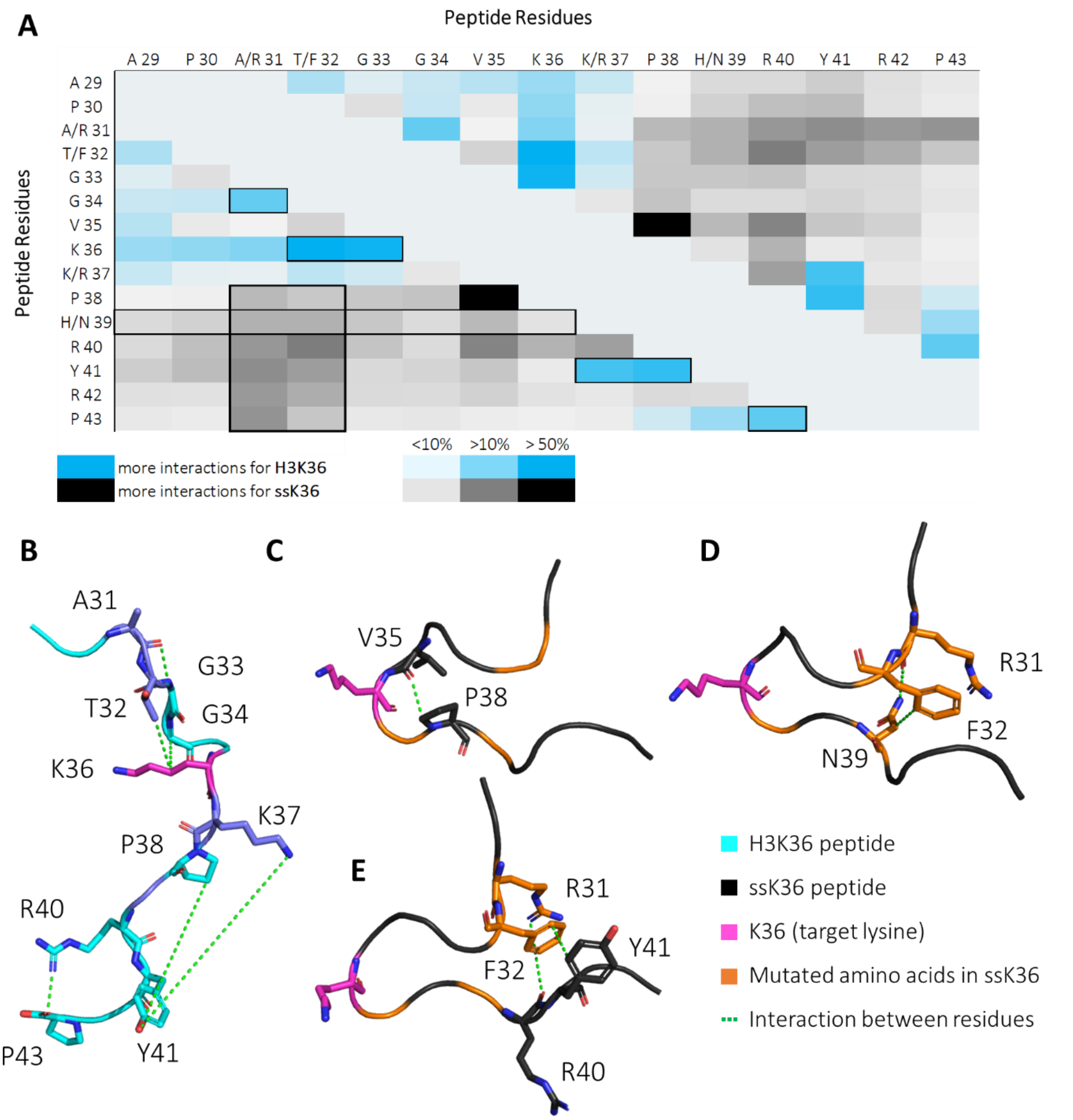

Supplementary Figure 2: Contact profiles of the H3K36 and ssK36 peptides display multiple conformational differences in solution. **A|** Contact frequency difference (4.5 Å cut-off) of simulations with H3K36 or ssK36 in a water box. The 4 amino acid differing between H3K36 and ssK36 (A/R31, T/F32, K/R37, H/N39) are engaged in different contacts, which cause a different conformational preference for ssK36. **B|** In H3K36, A31 preferably contacts G34, whereas T32 and G33 are in contact with K36. Additionally, K37 and P38 are in proximity to Y41. Moreover, residues R40 and P43 were observed in contact with each other. **C|** In ssK36, V35 and P38 are in close proximity. **D|** N39 of ssK36 is in contact with R31. **E|** The ssK36 specific residues R31 and F32 form contacts with Y41 and R40. The amino acids involved in contacts and K36 are shown as sticks, the ssK36 specific residues are coloured orange.

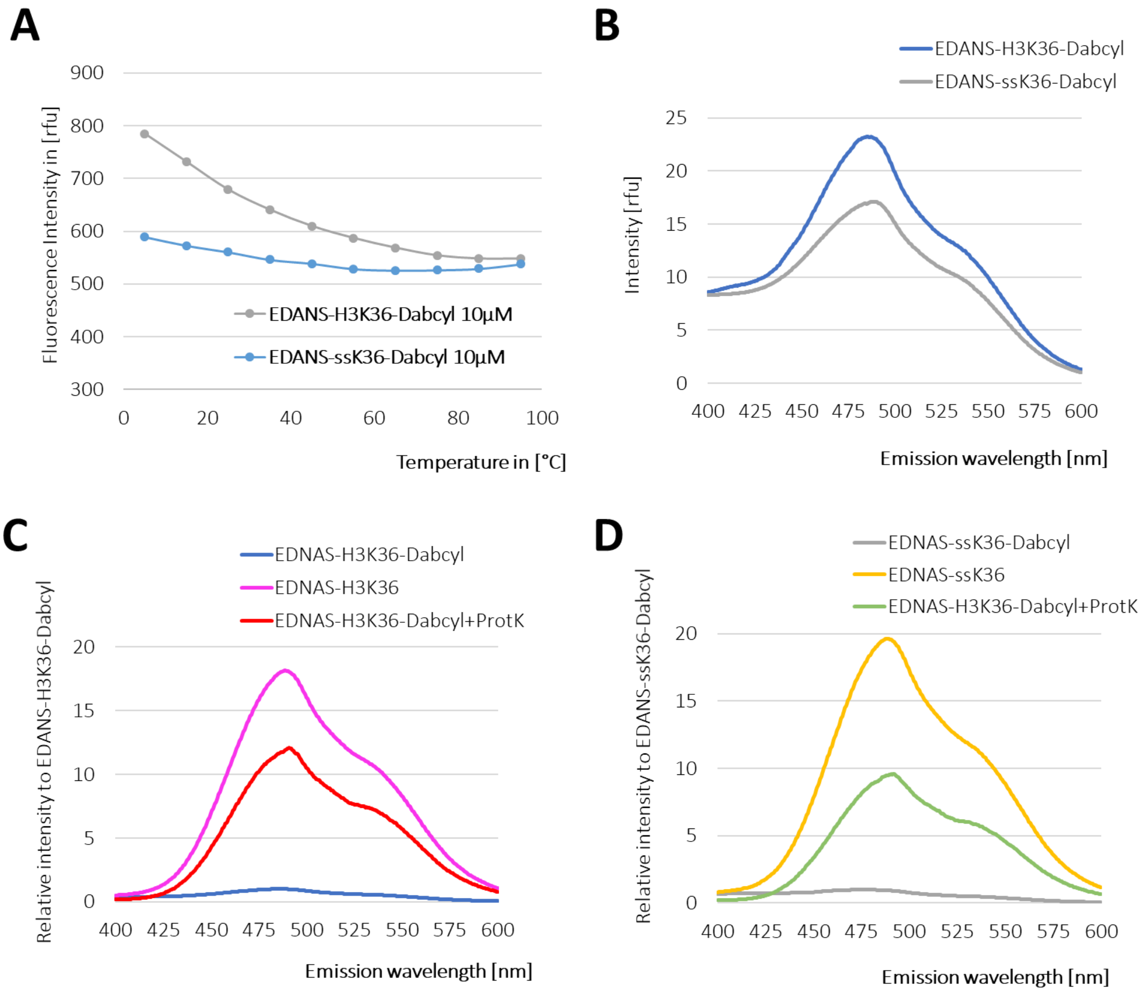

Supplementary Figure 3: Additional data related to the FRET experiments to analyse peptide conformations in solution. A| Emission at 490 nm of H3K36 and ssK36 with attached EDANS fluorophore at the N-terminus and the Dabcyl quencher at the C-terminus over a temperature gradient from 5°C to 95 °C. **B|** Fluorescence emission spectra of EDANS-H3K36-Dabcyl and EDANS-ssK36-Dabcyl. Excitation was at 340 nm and the emission was measured from 400-600 nm. Highest fluorescence intensity of both peptides was detected at 490 nm. The EDANS-H3K36-Dabcyl peptide showed a 1.35-fold higher intensity than EDANS-ssK36-Dabcyl. **C|** Fluorescence emission spectra of H3K36 peptide with and without quencher and of the Proteinase K digested peptide used in control experiments. **D|** Fluorescence emission spectra of ssK36 peptide with and without quencher and of the Proteinase K digested peptide used in control experiments.

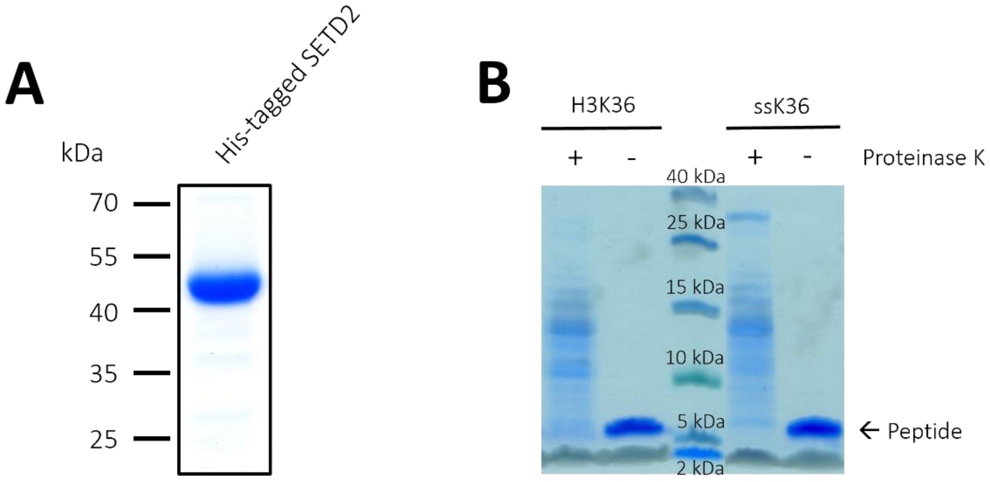

Supplementary Figure 4: Coomassie BB stained gel images of purified SETD2 enzyme and the H3K36 and ssK36 peptides. A| 16% SDS-PAGE showing the purified His-tagged SETD2 enzyme. **B|** 16% tricine gel of Proteinase K digested H3K36 and ssK36 peptides. Each peptide was digested for 1 h with Proteinase K. The gel picture shows one peptide band if no Proteinase K was added. After addition of Proteinase K, the peptide bands disappeared. The additional bands were part of the protease sample.

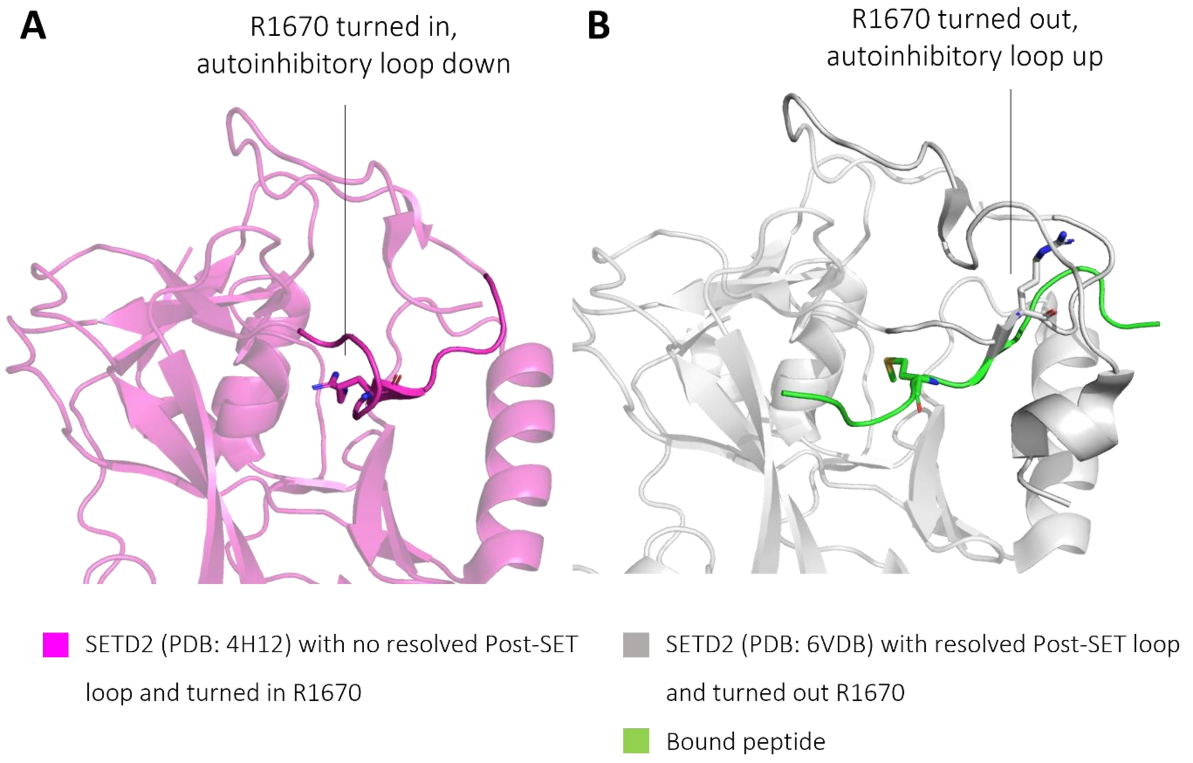

Supplementary Figure 5: Comparison of SETD2 structures with R1670 turned inwards and outwards. A| Crystal structure of SETD2 with R1670 turned inwards and the autoinhibitory loop in a closed conformation blocking the active site (PDB: 4H12). The post-SET loop is not resolved, indicating a high flexibility of this region with no bound peptide. **B|** Crystal structure of SETD2 with bound peptide (coloured green) and R1670 turned outwards (PDB: 6VDB). The autoinhibitory loop is moved up in an open conformation and the post-SET loop is resolved in front of the peptide.

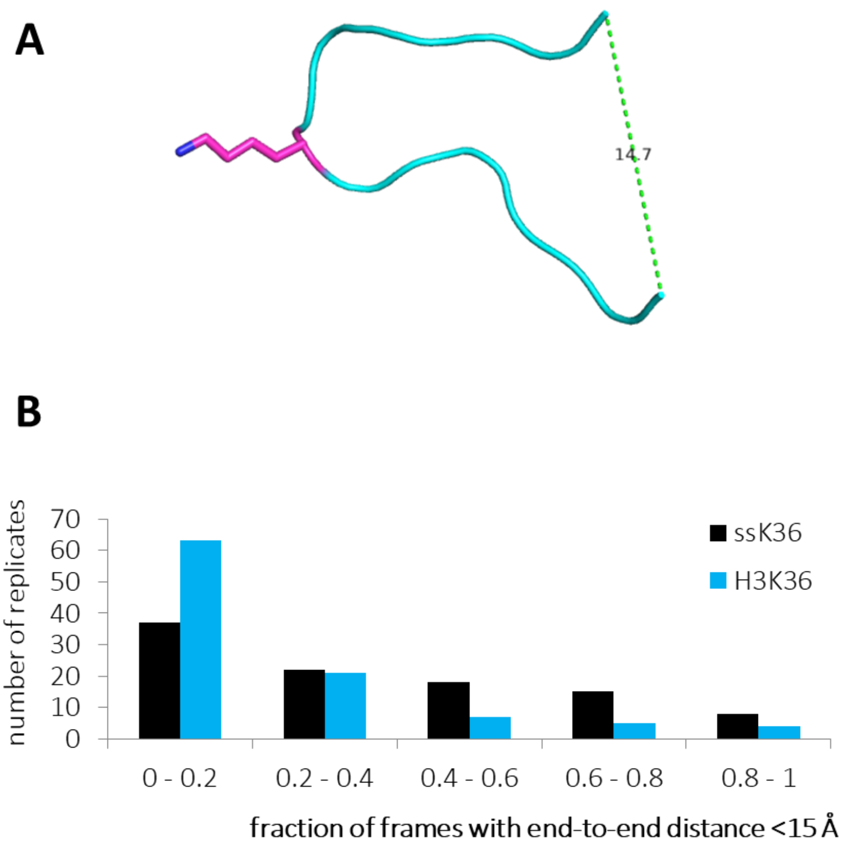

Supplementary Figure 6: Observation of peptides in hairpin conformation in the sMD experiments. **A|** In sMD experiments, hairpin conformations of the peptide are induced by pulling at lysine 36. The representative structure shown here has an end-to-end distance of 14.7 Å. **B|** Histogram showing the distribution of frames with end-to-end distance <15 Å. For each sMD simulation replicate the fraction of frames with an end-to-end distance <15 Å was determined and binned between 0 (no frames displayed this conformation) to 1 (all frames had an end-to-end distance <15 Å).

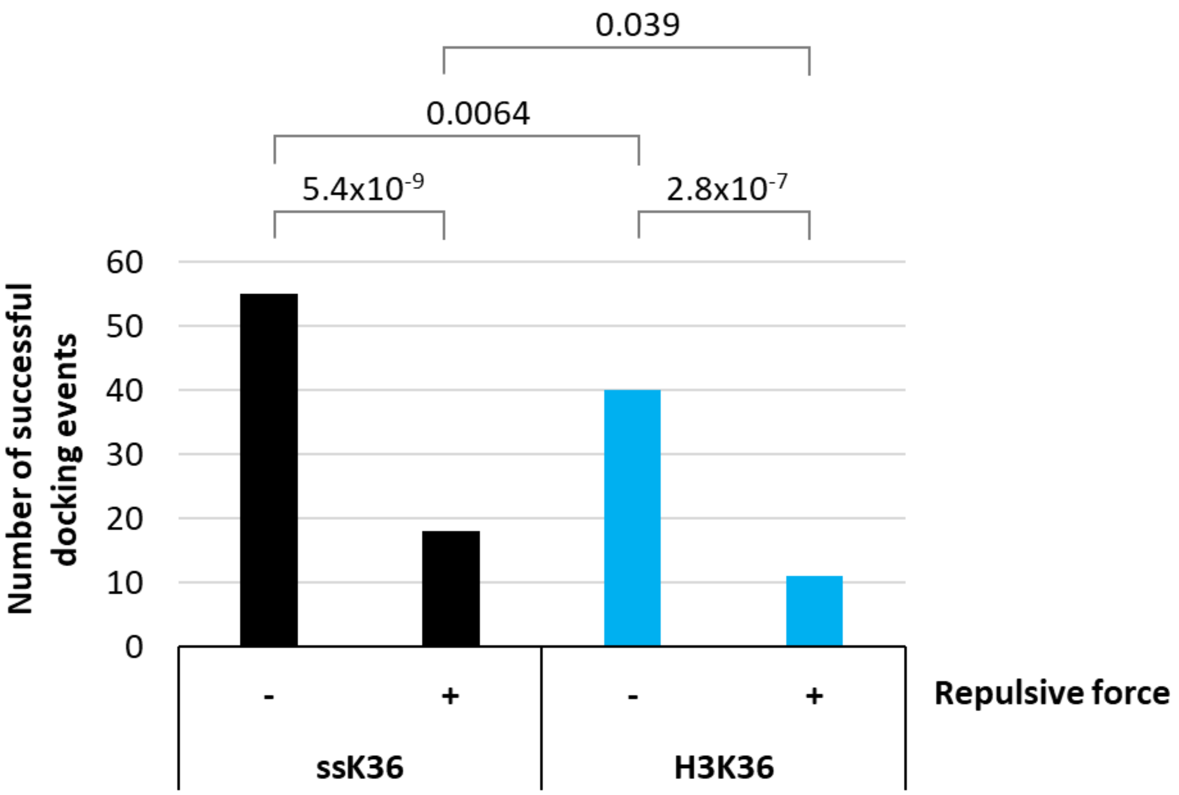

Supplementary Figure 7: Number of successful docking events in the sMD simulations and corresponding pairwise p-values. Pairwise p-values comparing the number of successful docking events of ssK36 and H3K36 with and without repulsive force were determined by binomial distribution against the null-hypothesis that both values were derived from one distribution.

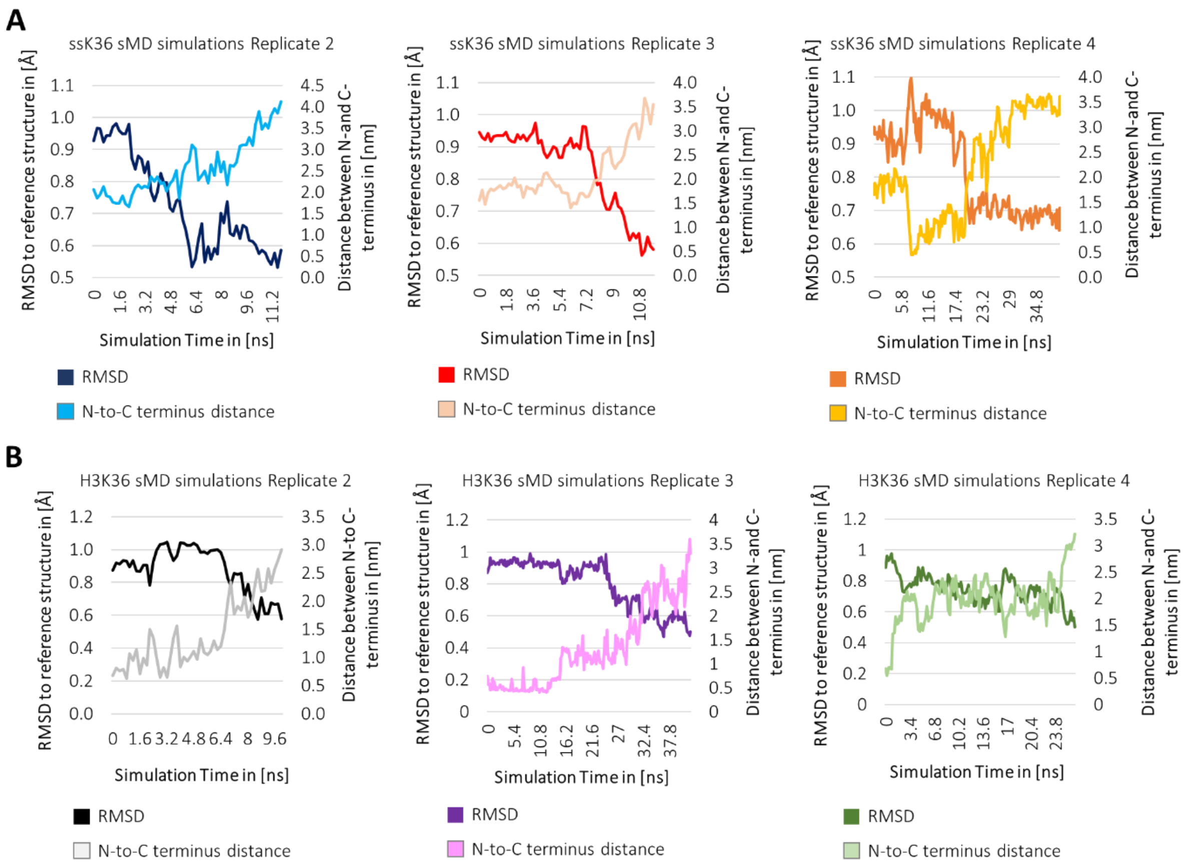

Supplementary Figure 8: H3K36 and ssK36 peptides unfold upon binding into the active site of SETD2. A-B| Additional representative replicates of successfully docked peptide association simulations. In general, the RMSD of the peptide conformation from the reference structure decreased while the N-C distance of the peptide increased. Shown are representative replicates from sK36 (A) and H3K36 (B) sMD simulations. The RMSD and N-to C-terminus distance was tracked until the minimum RMSD position was reached. The RMSD was calculated using the crystal structures of complexed peptides in SETD2 as references, after positional optimisation by rigid body movement (PDB: 5V21 for H3K36, PDB: 6VDB for ssK36). The N-C distances were calculated as the distance between the backbone nitrogen atoms of A29 and P43.

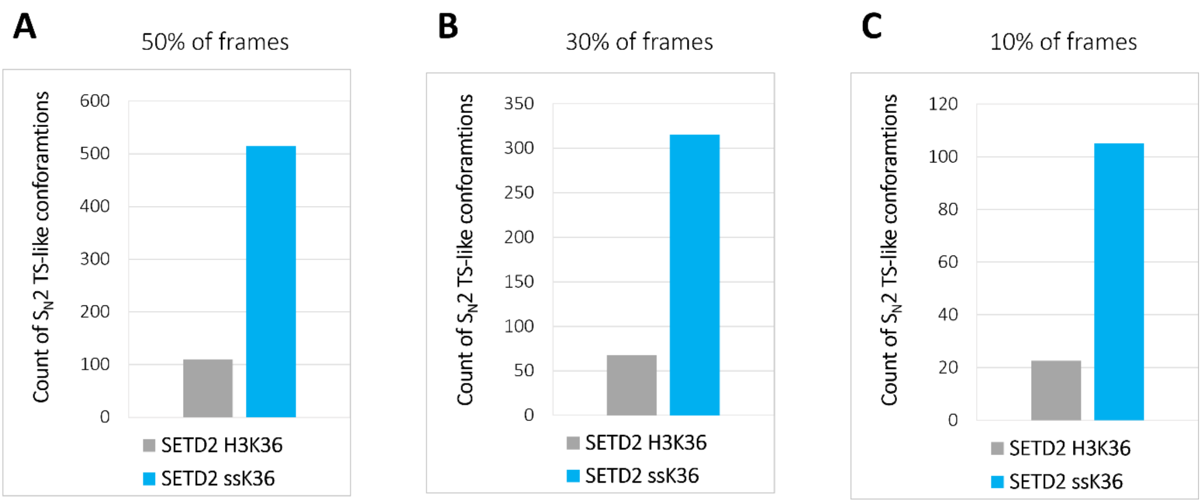

Supplementary Figure 9: Additional data related to MD simulations of peptide-SETD2 complexes. A-C| Subsets of data (50% = 2500 MD simulation frames per replicate, 30% = 1500 MD simulation frames per replicate, 10% = 500 MD simulation frames per replicate) were randomly selected and the number of S_N2 TS-like structures counted. Reducing the number of frames per replicate did not lead to noticeable changes in the ratio between H3K36 and ssK36.

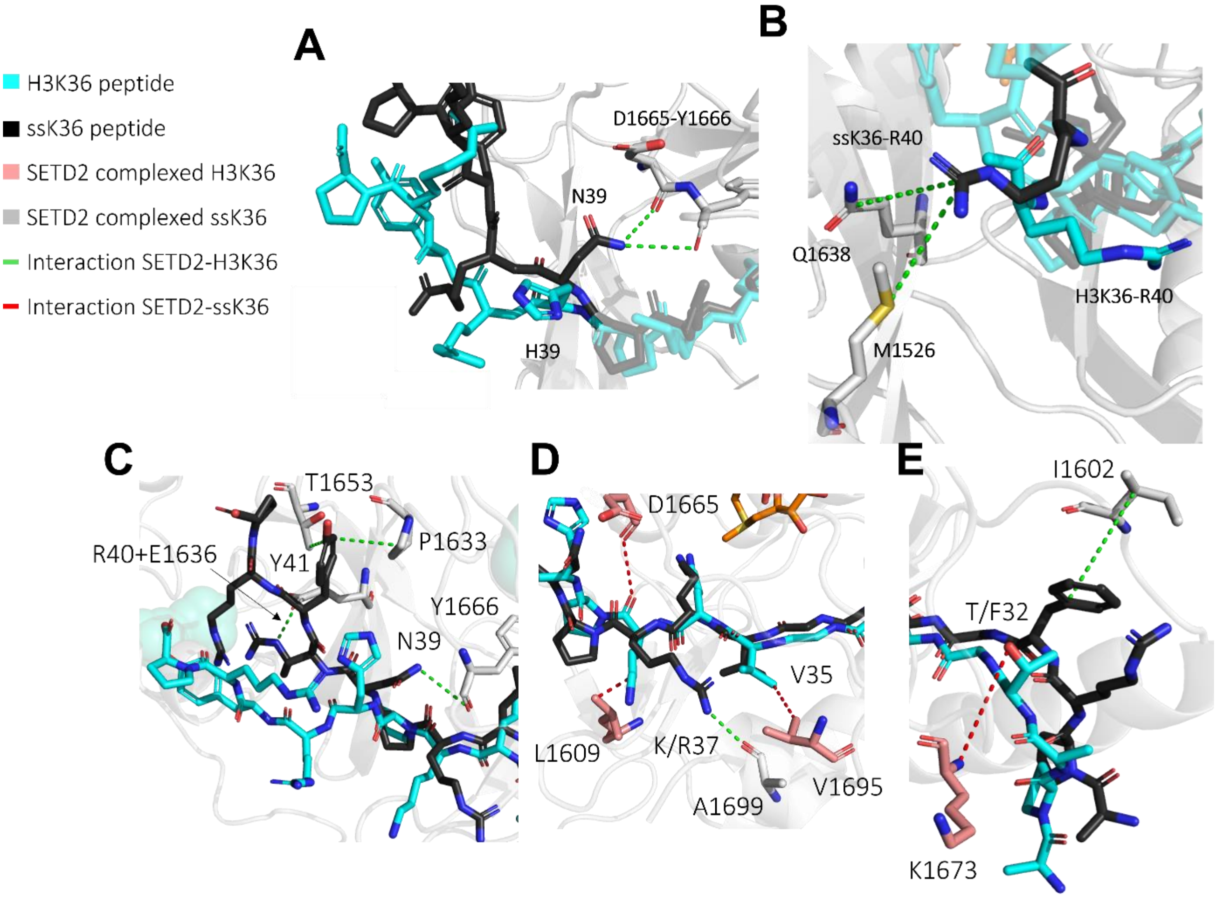

Supplementary Figure 10: Visualization of the contact profiles of H3K36 and ssK36 peptides bound to SETD2 observed in the MD simulations. A-E| Interaction differences (dashed red or green lines) of H3K36 and ssK36 identified in the contact profiles. Presented is an overlay of the H3K36 (coloured cyan) and ssK36 (coloured black) peptides complexed with SETD2 from representative MD simulations (coloured rose for SETD2 in complex with H3K36 and grey with ssK36, respectively).

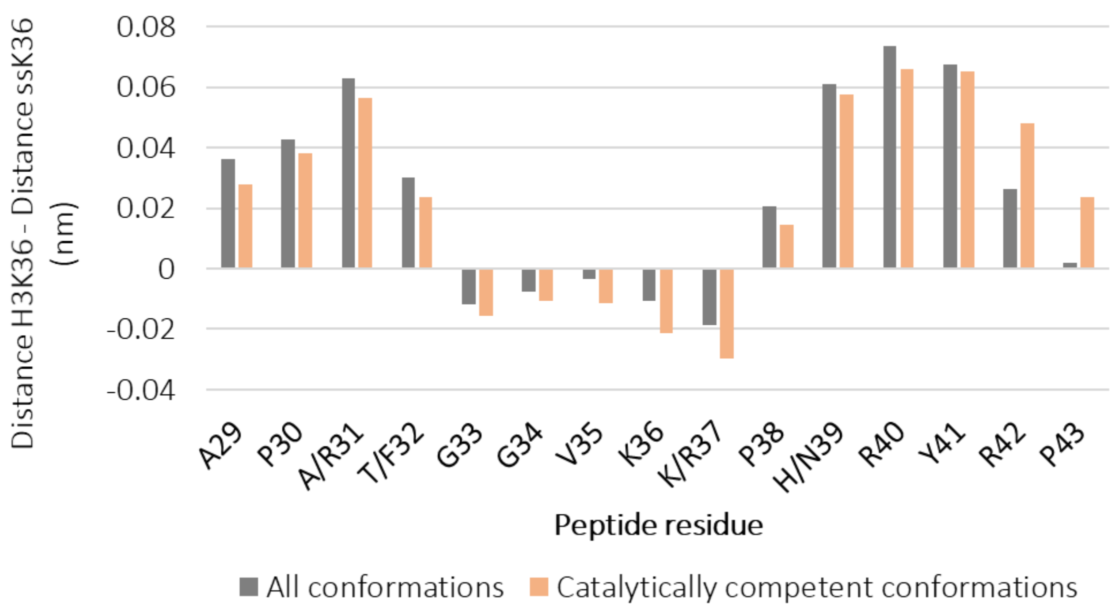

Supplementary Figure 11: Distance difference between the H3K36 and ssK36 peptide nitrogen backbone atoms and a reference point in the middle of SETD2 (R1625). Average distance of outer peptide residues A29-F32 and N39-P43 to the reference point was lower for ssK36 than for H3K36. In contrast, middle residues G33-K37 of H3K36 were closer to the reference point than for ssK36. Grey shading indicates that all conformations were used for calculation, orange shading indicates only catalytically competent conformations were considered.

Supplementary Tables

Supplementary Table 1: All atom RMSD comparison of centroid structures. The RMSD of distances between all atoms in the peptide conformation were calculated using PyMOL. The analysis shows that the extended conformation, preferably adopted by H3K36, and the hairpin conformation, preferred by ssK36, display the largest discrepancy.

All atom RMSD [Å]	Extended Conformation	Hairpin Conformation	Mixed Conformation
Extended Conformation	0	7.68	5.34
Hairpin Conformation	7.68	0	7.42
Mixed Conformation	5.34	7.42	0

Supplementary Table 2: Results of mutational studies of SETD2 residues involved in H3K36 and ssK36 peptide interaction.

Mutant	Effect of mutagenesis	Role in H3K36 and ssK36 interaction	Reference
Y1604 to A	Increased H3K36 methyltransferase activity	Interaction with F32/T32 and G33	Yang, et al. 2016 [1]
T1637 to A	Increased H3K36 methyltransferase activity	Interaction with H39/N39	Yang, et al. 2016 [1]
Y1666 to A	Strongly reduced H3K36 methyltransferase activity	Interaction with K36	Yang, et al. 2016 [1]
F1668 to A	Strongly reduced H3K36 methyltransferase activity	Interaction with V35 in both	Zheng et al, 2012 [2]; Yang, et al. 2016 [1]
Q1669 to A	Loss of H3K36 methyltransferase activity	Interaction with V35 and K/R37	Zheng et al, 2012 [2]
R1670 to A/V/L/I/F/P/W/K/Q	Reduced or lost H3K36 methyltransferase activity	Regulation of the autoinhibitory loop, Interaction with G33	Zheng et al, 2012 [2]
Y1671 to A	Strongly reduced H3K36 methyltransferase activity	Interaction with G33 and G34	Zheng et al, 2012 [2]; Yang, et al. 2016 [1]

Supplementary references

1 Yang, S. *et al.* Molecular basis for oncohistone H3 recognition by SETD2 methyltransferase. *Genes Dev* **30**, 1611-1616 (2016).

2 Zheng, W. *et al.* Sinefungin derivatives as inhibitors and structure probes of protein lysine methyltransferase SETD2. *J Am Chem Soc* **134**, 18004-18014 (2012).
