# Supplementary material for: Mechanistic basis of the increased methylation activity of the SETD2 protein lysine methyltransferase towards a designed super-substrate peptide
Source: Commun Chem. 2022 Oct 28;5:139. doi: 10.1038/s42004-022-00753-w (PMC9814698; doi:10.1038/s42004-022-00753-w)
Supplement: Supplementary file 1 — Jeltsch_PR File [file 42004_2022_753_MOESM1_ESM.pdf]

nature portfolio

Peer Review File

Mechanistic basis of the increased methylation activity of the SETD2 protein lysine methyltransferase towards a designed super-substrate peptide

Open Access This file is licensed under a Creative Commons Attribution 4.0 International License, which permits use, sharing, adaptation, distribution and reproduction in any medium or format, as long as you give appropriate credit to the original author(s) and the source, provide a link to the Creative Commons license, and indicate if changes were made. In the cases where the authors are anonymous, such as is the case for the reports of anonymous peer reviewers, author attribution should be to 'Anonymous Referee' followed by a clear attribution to the source work. The images or other third party material in this file are included in the article's Creative Commons license, unless indicated otherwise in a credit line to the material. If material is not included in the article's Creative Commons license and your intended use is not permitted by statutory regulation or exceeds the permitted use, you will need to obtain permission directly from the copyright holder. To view a copy of this license, visit http://creativecommons.org/licenses/by/4.0/.

Reviewers' comments:

Reviewer #1 (Remarks to the Author):

In this manuscript, Schnee and colleagues reported their computational and experimental efforts to mechanistically rationalize the increased activity of a designed substrate of SETD2. As reported in Ref 17, the authors identified a "super-substrate" of SETD2, ssK36, based on the collective information of how SETD2 interacts with the substrate peptide and tolerates individual amino acid residues. In the previous work, the authors reported ~100-fold increase of the activity of ssK36 in contrast of that of the wild-type K36 peptide. However, it remains puzzling how such increased activity can be achieved. The manuscript is for the mechanistic follow-up of the prior finding. SETD2 belongs to a large family of protein lysine methyltransferases (PKMTs) and a subfamily of H3K36 PKMT. SETD2 as well as many other PKMTs have been implicated in various developmental diseases and cancer. However, it is not clear how these enzymes recognize diverse substrates in particular non-histone targets. This work advanced such understanding from the perspective of the substrates, in particular their conformational dynamics to engage the interaction and transition-state assembling in the context of the PKMT enzyme. It is expected to be a key step to better understand enzymology of PKMTs. The reviewer recommends publishing this work after revising the manuscript and addressing the following concerns, in particular quantitative analysis and discussion of their computational results:

(1) The sufficiency and justification of the 50x70 ns. Although the computational power for the small 15-aa H3K36 and ssK36 peptides is not expected to be substantial, it is not clear whether the 50x70 ns are sufficient to capture all the important conformations of the two peptides. The authors should randomly select a subset of the data such as 50%, 30% and 10% and evaluate whether they are sufficient to lead to a similar conclusion for two clusters of conformations, an extended centroid structure and a hairpin-like bent conformation, and their probability distribution.
(2) The authors showed the temperature-dependent difference of the FRET signals of wt and ssH36. The puzzle here is that the hairpin-like bent conformation is expected to be enthalpy-driven and should be less popular at higher temperatures. In contrast, the extended centroid structure is expected to be entropy-driven and less popular at higher temperatures. Can the authors provide rationales about the independent and reverse tends for the two peptides?
(3) In terms of the computed ratios of the extended centroid structure versus a hairpin-like bent conformation, can the authors provide quantitative analysis and discussion of how this difference renders to the final of the ~100-fold increase of the activity of ssK36?
(4) Upon modeling the peptide association with SETD2, the authors started the SETD2 as an open conformation with a weak attractive force to better engage a SETD2-peptide complex and an additional repulsive force to the ends of the peptides. Presumably these maneuvers facilitate the assembling of the active transition state. However, even so, the authors only observed "3.6-fold (H3K36)" vs "2.8-fold (ssK36) decrease in docking efficiency" in comparison with the unconstrained settings. The reviewer' concerns lie in two aspects: the small difference brought by these modeling maneuvers and the possible to mask the true rate-limiting steps, which account for 100-fold increase of the activity of ssK36. The authors need to rule out these concerns.
(5) Given that the final conformation of the peptide substrate is an extended conformation, all the free-energy gain via the bent conformation prior to the transition state would be paid back eventually upon the formation of the SETD2-peptide complex, in which the peptide adapts an open conformation. It is clear how such paradox can be rationalized to give the 100-fold increase of the activity of ssK36.
(6) The overall analysis showed that the catalysis of SETD2 on methylation of wt and ssK36 peptides acts via multiple steps with specific preferences of the conformations. The prior report only showed the overall increase of apparent kcat/Km values. However, the contributions to the 100-fold increase of apparent kcat/Km can be readily dissected for kcat and Km experimentally. These values should be consistent with the analysis above. The authors should conduct such experiments to further strengthen their models.
(7) In Ref 17, the authors reported many single-point mutants with the altered substrate activities. The information can be used in this context to strength the current model. For instance, can authors

model, identify and cluster some mutants with the increase and decrease of the hairpin-like bent conformation and associate this observation with their substrate activities.

Collectively, the work described an interesting and sound modeling for SETD2 to catalyze its substrate methylation with multiple matched conformations in a stepwise manner. However, such observation is rather qualitative and correlative. The situation should be improved to solidify the current mechanism.

Reviewer #2 (Remarks to the Author):

In this manuscript, MD simulations combined with FRET assays were used to explore the driving factors for the rapid methylation of ssK36 peptide compared with wild-type H3K36 peptide. The conclusion partly explained the molecular mechanism of substrate recognition by SETD2. However, this manuscript mainly described the recognition and binding modes of ssK36 and SETD2. Considering that ssK36 was an artificially designed peptide, its biological significance was limited. A few points below should be addressed:
1. It was mentioned in this paper that ssK36 and H3K36 are more likely to form hairpin conformation in solvent, because of the intermolecular forces within mutant amino acids in ssK36 However, H3K36 itself could also form a certain hairpin conformation and thus access into SETD2. Although hairpin conformation occurred less frequently in H3K36, could H3K36 also have a weaker tendency to form hairpin? Considering that the methylation function of SETD2 has a certain sequence specificity and the preference of SETD2 for the hairpin structure in the conclusions of this paper, it is reasonable that H3K36 can also form the hairpin structure and this potential tendency should be discussed. In addition, more controls need to be introduced in this part, including point mutations of key amino acids of ssK36, random sequence polypeptides, etc.
2. Although SMD method could accelerate the processes to reach the target conformation, but in order to demonstrate the preference of SETD2 for the hairpin conformation, such method with repulsive force at both ends seemed to be artificial. Another peptide control, whose sequence was more difficult to form a hairpin conformation, was recommended, as long as more parallel experiments with scramble sequences.
3. It is obvious that a protein pocket with a certain shape could accommodate a smaller and aggregated peptide, but cannot hold an extended form due to steric effects. Therefore, it is recommended to specifically analyze relationship between the conformational preference and the pocket shape of SETD2 in the presence or absence of substrate polypeptides?
4. In this manuscript, interaction details of amino acids of SETD2 and two peptides during recognition were discussed. However, it was not complete to confirm these interactions only by simulation. Mutations on SETD2 at the in vitro molecular level and further verification carried out by biophysical assays were still needed.
5. In this manuscript, certain structural criterias were used to describe the methylation and transition states. However, it is well known that biological reactions are generally energy-driven. Whether this manuscript had focused on the energy variation during the binding and catalytic reactions? This would be more intuitively to describe the propensity of SETD2 for ssK36. By the way, more biophysical binding assays were recommended to evaluate the binding potential of different peptides with SETD2.
6. The amino acid interaction model proposed in this paper was limited to the difference between ssK36 and H3K36, and less focused on the biological function of SETD2. Further discussion of the relationship between key amino acids and biological functions needs to be introduced, such as the conservation of these sites within PHMT family or even species, the pathology characteristics and the potential role in the overall function of SETD2.
7. The analysis process of the kinetic simulation in this paper was limited to the substrate binding area. It is necessary to indicate whether SETD2 undergoes global conformational changes when it recognizes or catalyzes the substrate, and whether these changes may have a certain impact on the substrate preference.
8. Some figures were redundant, such as the repetition in the legends of Figures 8 and 9, which can

be merged.

Reviewer #3 (Remarks to the Author):

This manuscript describes mechanistic studies on the SETD2-catalyzed methylation of the histone H3-based super-substrate peptide ssK36. SETD2 belongs to a family of biomedically important histone lysine methyltransfereases that catalyze methylation of lysine residues in various proteins. The authors have computationally and experimentally investigated the mechanism of the ssK36 substrate superiority over the natural H3K36 sequence. Building on their previous report (ref. 17), the authors carried out molecular dynamics simulations of free ssK36/H3K36 peptides, their association processes with SETD2, and SETD2-ssK36/H3K36 complexes. In parallel, FRET studies provided the experimental support for the computational observations. The manuscript is well written and of good quality. The work will be appealing to enzymologists and chemical biologists, in particular those investigating molecular mechanisms of epigenetic processes. While somewhat interesting, the authors need to better describe the need to understand the mechanistic basis of the artificial ssK36 peptide by SETD2. What is the biological relevance to precisely investigate artificial peptides? What use better enzyme substrates could have?

Because detailed mechanistic studies of enzymes are often challenging, it is appreciated that the authors have tackled the SETD2-catalyzed methylation of histone H3 in detail. Each of the three proposed steps in this study could, however, be investigated further to provide more compelling evidence for difference in SETD2-catalyzed methylation of ssK36 and H3K36.

In addition to MD simulations and FRET analyses, free peptides could also be investigated in solution by NMR spectroscopy. This could be perhaps beyond the expertise of the authors, however, it would be valuable to examine both peptides by NMR spectroscopy to verify the potential hairpin conformation.

The association of ssK36/H3K36 peptides to SETD2 should be also investigated by ITC. This is particularly important because the conformational change in the binding process could be traced by the entropic term of free energy of binding. ITC binding work should be feasible, as only one enzyme and two peptides are needed. Similar binding studies have been recently used in examinations of SETD3 methyltransferase. If possible, SPR analyses would also be valuable to better understand the kinetics of the binding processes of ssK36 and H3K36. However, thermodynamic analyses using ITC are key, as they will reveal whether both peptides have comparable binding affinities and what is the difference in entropic and enthalpic terms.

Catalytically productive conformations in the SETD2-ssK36/H3K36 complexes could also be investigated further. QM/MM MD simulations can provide difference in free energy of methylation profiles between both peptides. This part can relate with kinetic analyses, testing whether the enhanced substrate efficiency is due to kcat and Km terms.

On page 15, the authors state that the post-SET loop was lifted upwards. How was this process done?

In Table 1, sequences show attachment of Dabcyl and Edans. These peptides were only used for FRET studies, but not for MD simulations.

In Abstract, the association reaction is mentioned. The association is a binding process, whereas reaction is conversion of substrate to product. The name ‘association reaction’ thus needs to be corrected.

Reviewer #4 (Remarks to the Author):

This manuscript reported the mechanistic studies of SETD2 towards a designed super-substrate peptide (ssK36) compared to the H3K36 peptide. Molecular dynamics (MD) simulations and biochemical FRET experiments were performed. The authors concluded ssK36 preferentially adopts a hairpin conformation in solution with V35 and K36 placed in the loop to access the active site of SETD2 and unfolds during the association reaction.

Although MD provides valuable insights into possible mechanisms, experimental data is essential to confirm those observations. Below are some concerns that need to be addressed.
1. The authors used FRET to confirm the proximity between two ends to support the claim of hairpin conformation observed in MD. However, FRET data did not directly support the hairpin conformation. Subsequent experiments will be needed for this claim. NMR study of the radiolabeled peptide with or without the addition of SETD2 would be a valuable experiment for validation.
2. Binding studies of V35 and K36 mutants with ssK36 along with wild type SETD2 and peptide to confirm their contributions.

Manuscript COMMSCHEM-22-0160

Schnee et al.: Mechanistic basis of the increased methylation activity of the SETD2 protein lysine methyltransferase towards a designed super-substrate peptide

Reply to all reviewers

Thank you very much for working with our manuscript and the constructive and helpful comments. Our paper provides evidence for a novel association pathway of peptides into SETD2, where binding of a peptide is preferred in a hairpin conformation. During the binding process, the hairpin unfolds and the peptide finally adopts the conformation seen in the crystal structures of SETD2-peptide complexes. We realized that all reviewers asked for additional experimental evidence to support our model. To this end, we conducted peptide methylation experiments with H3K36, ssK36 and variants thereof, which form a disulfide bond connecting the termini thereby introducing a stable hairpin conformation (**page 10-11, Figure 6D and E**). The observed reaction rates are summarized below:

H3K36 < H3K36(C-C) ≈ ssK36(C-C) < ssK36

Our finding that the hairpin stabilized H3K36(C-C) is methylated better than the original H3K36 clearly indicates that hairpin formation accelerates the reaction. However, the hairpin H3K36(C-C) and ssK36(C-C) are methylated at a reduced rate when compared with ssK36, indicating that the unfolding of the peptide during complex formation is beneficial for methylation. Finally, the observation that the hairpin stabilized forms of H3K36(C-C) and ssK36(C-C) are methylated at similar rates strongly supports the notion that the hairpin formation potential is the critical difference between both substrates. Hence, the results of this new experiment are in perfect agreement with our previous results and they provide strong additional evidence in favor of our model.

In addition, the manuscript has been modified at several places including the inclusion of additional data in response to the individual reviewers' comments. This includes:

- Analysis of the lifetimes of hairpin conformations of H3K36 and ssK36 (**page 6, Figure 2E**)
- Analysis of the lifetimes of TS-like conformations of H3K36- and ssK36-SETD2 complexes (**page 11, Figure 7D**)
- Clustering of peptide conformations with reduced data sets (**page 5, Supplementary Figure 1B-E**)
- Analysis of the formation of TS-like conformations with reduced data sets (**page 11, Supplementary Figure 9**)
- Better description of the final model and inclusion of a conceptual figure (**page 14, Figure 9**)
- Better explanation of the qualitative fit of the results of this paper to previous data (**page 15-16**)
- Better description of the modelling of SETD2 in an open conformation for the sMD analyses (**page 7-8 and 17**)

The details of these changes are described below. For convenience, larger changes in the manuscript are indicated in blue font in the version of the manuscript with marked changes. We believe that the paper has become significantly improved during the revision cycle.

Reply to the comments of the individual reviewers

Reviewer #1 (Remarks to the Author):

"In this manuscript, Schnee and colleagues reported their computational and experimental efforts to mechanistically rationalize the increased activity of a designed substrate of SETD2. As reported in Ref 17, the authors identified a "super-substrate" of SETD2, ssK36, based on the collective information of how SETD2 interacts with the substrate peptide and tolerates individual amino acid residues. In the previous work, the authors reported ~100-fold increase of the activity of ssK36 in contrast of that of the wild-type K36 peptide. However, it remains puzzling how such increased activity can be achieved. The manuscript is for the mechanistic follow-up of the prior finding. SETD2 belongs to a large family of protein lysine methyltransferases (PKMTs) and a subfamily of H3K36 PKMT. SETD2 as well as many other PKMTs have been implicated in various developmental diseases and cancer. However, it is not clear how these enzymes recognize diverse substrates in particular non-histone targets. This work advanced such understanding from the perspective of the substrates, in particular their conformational dynamics to engage the interaction and transition-state assembling in the context of the PKMT enzyme. It is expected to be a key step to better understand enzymology of PKMTs. The reviewer recommends publishing this work after revising the manuscript and addressing the following concerns, in particular quantitative analysis and discussion of their computational results:"

Reply: Thank you very much for the positive assessment.

"(1) The sufficiency and justification of the 50x70 ns. Although the computational power for the small 15-aa H3K36 and ssK36 peptides is not expected to be substantial, it is not clear whether the 50x70 ns are sufficient to capture all the important conformations of the two peptides. The authors should randomly select a subset of the data such as 50%, 30% and 10% and evaluate whether they are sufficient to lead to a similar conclusion for two clusters of conformations, an extended centroid structure and a hairpin-like bent conformation, and their probability distribution."

Reply: This analysis has been done as proposed and its results are described on **page 5 and in Suppl. Fig. 1B-E**. A similar analysis has also been conducted for the MD simulations of the peptide-SETD2 complexes (**page 11, Suppl. Fig. 9**). In both cases. the new data support the notion that the overall simulation time is sufficient to draw the conclusions.

"(2) The authors showed the temperature-dependent difference of the FRET signals of wt and ssH36. The puzzle here is that the hairpin-like bent conformation is expected to be enthalpy-driven and should be less popular at higher temperatures. In contrast, the extended centroid structure is expected to be entropy-driven and less popular at higher temperatures. Can the authors provide rationales about the independent and reverse tends for the two peptides?"

Reply: The reviewer is right. If we consider the equilibrium between the unfolded and hairpin peptide structures U $\rightleftharpoons$ H, the $\Delta H°'$ of hairpin formation should be <0 because of more interactions. At the same time $\Delta S°'$ is expected to be <0 as well, because of the reduced conformational freedom. If we

ignore the temperature dependence of these terms, one would expect that with increasing temperature the unfavorable ΔS term gains in impact and hence the hairpin should unfold at higher temperatures. This is exactly what we observed in the FRET experiments. We have explained this rationale more explicitly on **page 6** of the revised manuscript.

"(3) In terms of the computed ratios of the extended centroid structure versus a hairpin-like bent conformation, can the authors provide quantitative analysis and discussion of how this difference renders to the final of the ~100-fold increase of the activity of ssK36?"

Reply: Our study shows that the increased methylation of the ssK36 substrate when compared with H3K36 is based on two main effects: increased hairpin formation of ssK36, and better stabilization of the transition state conformation of the complex. Stimulated by the comments of the reviewer, we have expanded our analysis and now show that the ssK36 peptide also leads to hairpin conformations and TS-structures with increased lifetime. As shown in the new Figure panels 2E and 7D, long-lived hairpin structures are 4.8 more abundant with ssK36 and long-lived TS-like structures 30-fold. Hence, these effects can easily explain the overall 100-fold rate enhancement in the methylation of ssK36. This rationale is now described in the discussion section on **page 15**.

"(4) Upon modeling the peptide association with SETD2, the authors started the SETD2 as an open conformation with a weak attractive force to better engage a SETD2-peptide complex and an additional repulsive force to the ends of the peptides. Presumably these maneuvers facilitate the assembling of the active transition state. However, even so, the authors only observed "3.6-fold (H3K36)" vs "2.8-fold (ssK36) decrease in docking efficiency" in comparison with the unconstrained settings. The reviewer' concerns lie in two aspects: the small difference brought by these modeling maneuvers and the possible to mask the true rate-limiting steps, which account for 100-fold increase of the activity of ssK36. The authors need to rule out these concerns."

Reply: For a discussion of the 100-fold change in methylation rate, please see our response to the last question. Regarding the sMD data please note that the key point here is to show that docking is very difficult if hairpin formation is prevented (by the additional repulsive force). This observation holds for ssK36 and H3K36.

Unfortunately, this experiment is not suitable to reveal the real difference in the efficiencies of the docking of H3K36 and ssK36. With repulsive force, ssK36 cannot readily adopt the hairpin conformation, hence it loses its advantage. Without the repulsive force, the sMD approach using an attractive force stimulates hairpin formation of H3K36 and ssK36. Hence under these conditions, the disadvantage of H3K36 is compensated. At this point unconstrained MD simulations of the association reaction would be required, which currently are beyond the state of the art.

"(5) Given that the final conformation of the peptide substrate is an extended conformation, all the free-energy gain via the bent conformation prior to the transition state would be paid back eventually upon the formation of the SETD2-peptide complex, in which the peptide adapts an open conformation. It is clear how such paradox can be rationalized to give the 100-fold increase of the activity of ssK36."

Reply: Please note that our data (and the new experimental findings described on **page 10**) clearly indicate that both substrates transiently adopt a hairpin conformation during the association reaction. Hence resolution of this structure is needed in both cases. In case of ssK36, the hairpin is stabilized, but it also forms more and stronger contacts to the enzyme as shown in our previous crystal structure analysis (Schuhmacher et al., 2020) and now also in our MD simulations.

"(6) The overall analysis showed that the catalysis of SETD2 on methylation of wt and ssK36 peptides acts via multiple steps with specific preferences of the conformations. The prior report only showed the overall increase of apparent kcat/Km values. However, the contributions to the 100-fold increase of apparent kcat/Km can be readily dissected for kcat and Km experimentally. These values should be consistent with the analysis above. The authors should conduct such experiments to further strengthen their models."

Reply: We like to mention, that the multistep, multifactor effects observed here cannot be easily connected with kcat and Km values. However, in the previous paper (Schuhmacher et al., 2020), we showed that the Km of ssK36 is only marginally improved (1.4-fold), which leaves the largest part of the 100-fold increase in reaction rate to the kcat term. This is in very good agreement with our findings that kinetic barriers during the association reaction are lowered for ssK36, and its shows improved ability to stabilize transition state like conformations of the SETD2-peptide complex. This important explanation has been included in the discussion of the revised manuscript on **page 15**.

"(7) In Ref 17, the authors reported many single-point mutants with the altered substrate activities. The information can be used in this context to strength the current model. For instance, can authors model, identify and cluster some mutants with the increase and decrease of the hairpin-like bent conformation and associate this observation with their substrate activities."

Reply: Thank you for this insightful recommendation. We have inspected the literature and made the interesting finding, that mutation of peptide interacting residues led to strong reductions in activity, if peptide residues conserved between H3K36 and ssK36 are concerned, highlighting the essential roles of these interactions. However, mutations of Y1604 and T1637, which both contact residues that are altered in ssK36, led to an increase in H3K36 methylation, indicating that these regions are not ideal for catalysis in H3K36. This information has been described in the discussion of the revised manuscript on **page 12**.

"Collectively, the work described an interesting and sound modeling for SETD2 to catalyze its substrate methylation with multiple matched conformations in a stepwise manner. However, such observation is rather qualitative and correlative. The situation should be improved to solidify the current mechanism."

Reply: Thank you for the helpful comments pointing towards weaknesses of our manuscript. We are optimistic that the revised version of the paper convincingly addresses most of your points.

Reviewer #2 (Remarks to the Author):

"In this manuscript, MD simulations combined with FRET assays were used to explore the driving factors for the rapid methylation of ssK36 peptide compared with wild-type H3K36 peptide. The conclusion partly explained the molecular mechanism of substrate recognition by SETD2. However, this manuscript mainly described the recognition and binding modes of ssK36 and SETD2. Considering that ssK36 was an artificially designed peptide, its biological significance was limited. A few points below should be addressed:

1. It was mentioned in this paper that ssK36 and H3K36 are more likely to form hairpin conformation in solvent, because of the intermolecular forces within mutant amino acids in ssK36 However, H3K36 itself could also form a certain hairpin conformation and thus access into SETD2. Although hairpin conformation occurred less frequently in H3K36, could H3K36 also have a weaker tendency to form hairpin? Considering that the methylation function of SETD2 has a certain sequence specificity and the preference of SETD2 for the hairpin structure in the conclusions of this paper, it is reasonable that H3K36 can also form the hairpin structure and this potential tendency should be discussed. In addition, more controls need to be introduced in this part, including point mutations of key amino acids of ssK36, random sequence polypeptides, etc."

Reply: Our sMD data show that the hairpin is an essential transient state during peptide binding to SETD2. The reviewer is right in remarking that H3K36 also can form a hairpin structure, this we do observe in solution MD simulation (Fig. 2) and in the sMD simulations (Fig. 4). However, ssK36 forms the hairpin more readily which provides one contribution to its enhanced methylation rate. The direct contribution of the hairpin structure to peptide methylation is clearly shown in our new kinetics data explained in the response to all reviewers.

"2. Although SMD method could accelerate the processes to reach the target conformation, but in order to demonstrate the preference of SETD2 for the hairpin conformation, such method with repulsive force at both ends seemed to be artificial. Another peptide control, whose sequence was more difficult to form a hairpin conformation, was recommended, as long as more parallel experiments with scramble sequences."

Reply: Please note that other unrelated or scrambled peptides cannot be used, as they will not bind to SETD2 and not be methylated. However, we understood that the reviewer requests additional experimental evidence to support our model. This is why we conducted the peptide methylation experiments described above in the reply to all reviewers. In these experiments, a hairpin is artificially introduced into the H3K36 and ssK36 peptides by a disulfide bond connecting their ends. Hence, the methylation of the regular and hairpin-enforced peptides can be studied directly side-by-side, which addresses the requests made in this point of the reviewer. As described above, the results of these methylation reaction are in perfect agreement with critical details of our model.

"3. It is obvious that a protein pocket with a certain shape could accommodate a smaller and aggregated peptide, but cannot hold an extended form due to steric effects. Therefore, it is recommended to specifically analyze relationship between the conformational preference and the pocket shape of SETD2 in the presence or absence of substrate polypeptides?"

Reply: The peptide binding site of SETD2 is better described as a cleft. This has been clarified in the revised manuscript at several places. Our model is that the binding cleft will be partially closed in the absence of peptide. The hairpin conformation with the V35 and the target K36 at the tip of the hairpin can directly dock into the hydrophobic binding patch of SETD2 if this is exposed. This contact represents the most important anchor point for binding. Then, the peptide can unfold and the binding cleft open up stepwise to form the other specific contacts in a zipper like fashion. In contrast to this, an extended peptide would need a fully open binding cleft for V35/K36 to reach the hydrophobic contact point without having steric clashes with surrounding residues. This concept has been described more explicitly in the discussion of the revised manuscript on **page 14** and we added a conceptual figure to illustrate the model (**Figure 9**).

"4. In this manuscript, interaction details of amino acids of SETD2 and two peptides during recognition were discussed. However, it was not complete to confirm these interactions only by simulation. Mutations on SETD2 at the in vitro molecular level and further verification carried out by biophysical assays were still needed."

Reply: We understand that the reviewer is requesting experimental evidence for our model. This is now provided in the methylation kinetics described in the reply to all reviewers. The new data directly confirm key elements of our model i.e. the hairpin assisted association and the unfolding of the hairpin during the binding process.

"5. In this manuscript, certain structural criterias were used to describe the methylation and transition states. However, it is well known that biological reactions are generally energy-driven. Whether this manuscript had focused on the energy variation during the binding and catalytic reactions? This would be more intuitively to describe the propensity of SETD2 for ssK36. By the way, more biophysical binding assays were recommended to evaluate the binding potential of different peptides with SETD2."

Reply: Enzyme catalysis is based on the stabilization of the transition state. This property was directly tested in sMD and MD simulations, because the propensity to adopt a Transition state (TS)-like conformation in the MD simulations is directly related to its Gibbs energy (ΔG) via the Boltzmann equation. Criteria were only used to describe a particular conformation as being TS-like in case of peptide-SETD2 complexes. The geometric criteria used for this are beyond doubt as they were directly derived from the well-known geometry of the S_N2 transition state of the lysine methylation reaction.

"6. The amino acid interaction model proposed in this paper was limited to the difference between ssK36 and H3K36, and less focused on the biological function of SETD2. Further discussion of the relationship between key amino acids and biological functions needs to be introduced, such as the conservation of these sites within PHMT family or even species, the pathology characteristics and the potential role in the overall function of SETD2."

Reply: The focus of our work was on the understanding of the mechanism behind the increased methylation rate of ssK36. All peptide interacting residues in this part of the SET domain of SETD2 are fully conserved in an alignment of 64 representative SETD2 homologs from mammals. This information is now provided in the manuscript on **page 12.**

"7. The analysis process of the kinetic simulation in this paper was limited to the substrate binding area. It is necessary to indicate whether SETD2 undergoes global conformational changes when it recognizes or catalyzes the substrate, and whether these changes may have a certain impact on the substrate preference."

Reply: Such an analysis would require long unconstrained MD simulations of the association process of peptides to SETD2 in several replicates. This is clearly beyond the scope of our work and at the edge of technical feasibility.

"8. Some figures were redundant, such as the repetition in the legends of Figures 8 and 9, which can be merged."

Reply: Thank you for this hint. We combined the original Figures 8 and 9 and moved some contents to the supplement. Please note that the original Fig. 8 and 9 (now Fig. 8A and B) did show different things and the figures are also differed from one another. Fig. 8A shows the overall structural analysis while Fig. 8B is based on the TS-like conformations more likely to be relevant for catalysis.

Reviewer #3 (Remarks to the Author):

(1) "This manuscript describes mechanistic studies on the SETD2-catalyzed methylation of the histone H3-based super-substrate peptide ssK36. SETD2 belongs to a family of biomedically important histone lysine methyltransfereases that catalyze methylation of lysine residues in various proteins. The authors have computationally and experimentally investigated the mechanism of the ssK36 substrate superiority over the natural H3K36 sequence. Building on their previous report (ref. 17), the authors carried out molecular dynamics simulations of free ssK36/H3K36 peptides, their association processes with SETD2, and SETD2-ssK36/H3K36 complexes. In parallel, FRET studies provided the experimental support for the computational observations. The manuscript is well written and of good quality. The work will be appealing to enzymologists and chemical biologists, in particular those investigating molecular mechanisms of epigenetic processes. While somewhat interesting, the authors need to better describe the need to understand the mechanistic basis of the artificial ssK36 peptide by SETD2. What is the biological relevance to precisely investigate artificial peptides? What use better enzyme substrates could have?"

Reply: Thank you very much for the positive statements that our "manuscript is well written and of good quality" and the "work will be appealing to enzymologists and chemical biologists, in particular those investigating molecular mechanisms of epigenetic processes."

Our research provides novel mechanistic insights that may for example help in the development of novel PKMT inhibitors. Moreover, we provide novel insight into the mechanism of a very important enzymatic reaction in human cells. This information is now provided in the abstract, introduction and discussion of the revised manuscript on **pages 2, 4, and 16**.

(2) "Because detailed mechanistic studies of enzymes are often challenging, it is appreciated that the authors have tackled the SETD2-catalyzed methylation of histone H3 in detail. Each of the three proposed steps in this study could, however, be investigated further to provide more compelling evidence for difference in SETD2-catalyzed methylation of ssK36 and H3K36."

Reply: We understand that the reviewer is requesting additional experimental evidence for our model. This is now provided in the methylation kinetics described in the reply to all reviewers. The new data directly confirm key elements of our model i.e. the hairpin assisted association and the unfolding of the hairpin during the binding process.

(3) "In addition to MD simulations and FRET analyses, free peptides could also be investigated in solution by NMR spectroscopy. This could be perhaps beyond the expertise of the authors, however, it would be valuable to examine both peptides by NMR spectroscopy to verify the potential hairpin conformation."

Reply: We like to mention that our FRET analyses directly probe the end-to-end distance of the peptide, which is the essential parameter of interest in the context of our work. NMR would be very complicated, an isotope labelled peptide would be needed, all resonances would need to be assigned and most of the information provided by NMR would not be directly related to the topic of our work.

(4) "The association of ssK36/H3K36 peptides to SETD2 should be also investigated by ITC. This is particularly important because the conformational change in the binding process could be traced by the entropic term of free energy of binding. ITC binding work should be feasible, as only one enzyme and two peptides are needed. Similar binding studies have been recently used in examinations of SETD3 methyltransferase. If possible, SPR analyses would also be valuable to better understand the kinetics of the binding processes of ssK36 and H3K36. However, thermodynamic analyses using ITC are key, as they will reveal whether both peptides have comparable binding affinities and what is the difference in entropic and enthalpic terms."

Reply: The key method of studying enzyme mechanisms are kinetic assays, because this is the only approach to probe the transition state properties of the reaction. Hence our novel data described in the reply to all reviewers are most suitable to provide experimental validation of our kinetic model. In principle, it is difficult to study enzymatic mechanisms by ITC, which is an equilibrium binding method. SPR provides some kinetic resolution of the association reaction, but not in the required time scale and the association rate constants determined by SPR are often limited by mass transfer during diffusion.

(5) "Catalytically productive conformations in the SETD2-ssK36/H3K36 complexes could also be investigated further. QM/MM MD simulations can provide difference in free energy of methylation profiles between both peptides. This part can relate with kinetic analyses, testing whether the enhanced substrate efficiency is due to kcat and Km terms."

Reply: The QM/MM experiments would be interesting, but they are clearly beyond the scope of the current manuscript. The Michaelis-Menten experiments have already been reported in our previous paper (Schuhmacher et al., 2020), where we showed that the Km of ssK36 is only marginally improved (1.4-fold), which leaves the largest part of the 100-fold increase in reaction rate to the kcat term. This is in very good agreement with our findings that kinetic barriers during the association reaction are lowered for ssK36, and its shows improved ability to stabilize transition state like conformations of the SETD2-peptide complex. This important explanation has been included in the discussion section of the revised manuscript on **page 15**.

(6) "On page 15, the authors state that the post-SET loop was lifted upwards. How was this process done?"

Reply: This modelling step has been described in detail on **page 7 and 17** of the revised manuscript.

(7) "In Table 1, sequences show attachment of Dabcyl and Edans. These peptides were only used for FRET studies, but not for MD simulations."

Reply: It has been clarified now, that the information refers to the peptides used in the biochemical experiments.

(8) "In Abstract, the association reaction is mentioned. The association is a binding process, whereas reaction is conversion of substrate to product. The name 'association reaction' thus needs to be corrected."

Reply: Wording has been changed.

Reviewer #4 (Remarks to the Author):

"This manuscript reported the mechanistic studies of SETD2 towards a designed super-substrate peptide (ssK36) compared to the H3K36 peptide. Molecular dynamics (MD) simulations and biochemical FRET experiments were performed. The authors concluded ssK36 preferentially adopts a hairpin conformation in solution with V35 and K36 placed in the loop to access the active site of SETD2 and unfolds during the association reaction.

Although MD provides valuable insights into possible mechanisms, experimental data is essential to confirm those observations. Below are some concerns that need to be addressed."

Reply: Thank you for concluding that our paper provides "valuable insights". We understand that the reviewer is requesting experimental evidence for our model. This is now provided in the methylation kinetics described in the reply to all reviewers. The new data directly confirm key elements of our model i.e. the hairpin assisted association and the unfolding of the hairpin during the binding process.

"1. The authors used FRET to confirm the proximity between two ends to support the claim of hairpin conformation observed in MD. However, FRET data did not directly support the hairpin conformation. Subsequent experiments will be needed for this claim. NMR study of the radiolabeled peptide with or without the addition of SETD2 would be a valuable experiment for validation."

Reply: We like to mention that our FRET analyses directly probe the end-to-end distance of the peptide, which is the essential parameter of interest in the context of our work. NMR would be very complicated, an isotope labelled peptide would be needed, all resonances would need to be assigned and most of the information provided by NMR would not be directly related to the topic of our work. Moreover, NMR in presence of SETD2 will not be possible, as the protein cannot be purified at the concentrations required for NMR.

"2. Binding studies of V35 and K36 mutants with ssK36 along with wild type SETD2 and peptide to confirm their contributions."

Reply: As mentioned above, we provided additional and very strong experimental evidence in favor of our model. The important role of V35 for SETD2 catalysis has been demonstrated previously and this information is provided on page 15 of the revised manuscript. Moreover, comparison of methylated data with K36 containing H3K36 and ssK36 and enzyme inhibition data by the corresponding K36M peptides indicates that K36 itself has an important role in the interaction of SETD2 with the ssK36 peptide. This information is also provided on p. 15 of the revised manuscript.

REVIEWERS' COMMENTS:

Reviewer #1 (Remarks to the Author):

My previous concerns have been largely addressed upon the revision. The reviewer suggests publishing this work.

Reviewer #3 (Remarks to the Author):

The authors have been responsive to earlier suggestions and have significantly improved the manuscript by adding new important computational and experimental data. While additional binding experiments could have also been carried out to strengthen the mechanistic insight of SETD2 catalysis, the work in general provides strong evidence for the role of the hairpin conformation of H3K36 peptide in productive SETD2 catalysis. I view the manuscript to be acceptable for publication.

Manuscript COMMSCHEM-22-0160A

Schnee et al.: Mechanistic basis of the increased methylation activity of the SETD2 protein lysine methyltransferase towards a designed super-substrate peptide

Reviewer reply

There were no open questions left. We like to thank all reviewers for working with our manuscript and the constructive and helpful comments.
